# Reduced Risk of Recurrent Fragility Fractures After a Primary Care–Based Fracture Prevention Intervention: A 20-Year Non-Randomized Controlled Follow-Up Study in Women Aged 70–100

**DOI:** 10.1080/02813432.2025.2571929

**Published:** 2025-11-06

**Authors:** Moses Sjölander, Lisa Alvunger, Robert Eggertsen, Anna Lindgren, Ulrica Mölstad, Ferdinando Petrazzuoli, Anna Segernäs, Hans Thulesius, Pär Wanby, Daniel Albertsson

**Affiliations:** ^a^ Department of Health, Medicine and Caring Sciences, Linköping University, Linköping, Sweden; ^b^ Region Västra Götaland, Research, Education, Development and Innovation, Primary Health Care, Gothenburg, Sweden; ^c^ Department of Public Health and Community Medicine, Sahlgrenska Academy, University of Gothenburg, Gothenburg, Sweden; ^d^ Centre for Mathematical Sciences, Lund University, Lund, Sweden; ^e^ Department of Research & Development, Region Kronoberg, Växjö, Sweden; ^f^ School of Specialization in Community Medicine and Primary Care, University of Naples “Federico II”, Naples, Italy; ^g^ Center for Primary Health Care Research, Department of Clinical Sciences, Lund University, Malmö, Sweden; ^h^ Primary Health Care Centre Ekholmen, Linköping and Department of Health, Medicine and Caring Sciences, Linköping University, Linköping, Sweden; ^i^ Department of Medicine and Optometry, Linnaeus University, Kalmar, Sweden; ^j^ Department of Internal Medicine, Section of Endocrinology, Region Kalmar County, Sweden

**Keywords:** Primary health care, fragility fractures, hip fractures, fracture prevention, risk factors, longitudinal studies, osteoporosis

## Abstract

**Background:**

In Sweden 70,000 people suffer fragility fractures annually, including 16,000 hip fractures with one-year mortality of up to 25%. Strategies to prevent falls, improve physical function, and enhance bone strength have shown mixed results.

**Aim:**

To evaluate the incidence of hip and other fragility fractures following a fracture prevention intervention and assess baseline risk factors for long-term fracture outcomes.

**Methods:**

1,233 rural Swedish women aged 70–100 years in 2002 were followed until 2021 after a primary care-based, non-randomized graded fracture prevention intervention 2002–2004 that included physical activity, fall prevention, and pharmacological treatment tailored to hip fracture risk. Fractures were identified through radiology reports 2002–2021.

**Results:**

The most common fractures occurred in the hip with 236 women sustaining 268 hip fractures with highest incidence in women aged 90–94 years. One-year hip fracture mortality was 27%. Hip fractures occurred in 17.7% of the intervention group (77/434) and 19.9% of controls (159/799, *p* = 0.36). Repeated fragility fractures occurred in 14.1% of the intervention group and 18.6% of controls (OR 0.71; 95% CI 0.53–1.0, *p* = 0.047), particularly when one fracture involved the hip (OR 0.54 (95% CI 0.31–0.95), *p* = 0.037). Increasing age (HR 1.8–4.0), height >167 cm (HR 1.6; 95% CI 1.1–2.2), and weight <60 kg (HR 1.5; 95% CI 1.1–2.0) were significant baseline risk factors.

**Conclusions:**

We noticed a non-significant reduction in hip fractures after 20 years, yet repeated fractures were less frequent in the intervention group suggesting a potential long-term benefit. Older, taller and lighter women were at greater risk for hip fracture.

## Introduction

Fragility fractures represent a significant and growing public health concern worldwide among the older population. These fractures, commonly involving the hip, wrist, vertebrae, humerus, and pelvis, are associated with substantial morbidity, reduced quality of life, and increased healthcare costs. The global burden of fragility fractures is expected to rise significantly by 2030 due to demographic shifts and increasing life expectancy [1,2].

A European study estimated that disability-adjusted life years (DALYs) lost due to fragility fractures surpass those associated with several major non-communicable diseases, including stroke and chronic obstructive pulmonary disease [1]. Among these fractures, hip fractures are the most severe, often resulting in hospitalization, surgery, and long-term functional impairment [1]. Sweden and other Nordic countries have among the highest hip fracture incidences globally, with approximately 70,000 fragility fractures occurring annually in Sweden, including 16,000 hip fractures. Of these, 66% affect women and 34% men [3,4]. The remaining lifetime risk of suffering a hip fracture for Swedish women aged 50 years and older was estimated at 22.8% [1].

In Sweden one-year mortality rate following a hip fracture was 20% for women and 33% for men, with no significant reduction observed over two decades [5].

### Fracture-related risk factors

Several risk factors contribute to fragility fractures, particularly among postmenopausal women with osteoporosis or a history of previous fractures. These include advanced age, medical conditions, pharmacological therapies (e.g. glucocorticoids), previous falls, and low bone mineral density [6]. Additional risk factors include maternal history of hip fracture, inability to rise from a chair without arm support, low body weight compared to one's weight at age 25 [7,8], and tall stature [9]. Low physical activity and impaired muscle function are also independently associated with an increased risk of fragility fractures, irrespective of bone mineral density [10].

A recent fracture further elevates the risk of subsequent fractures, particularly within the first 12 to 24 months following the initial event [2]. Importantly, tools such as the World Health Organization's Fracture Risk Assessment Tool (FRAX) may underestimate both initial and recurrent fracture risk, especially among those with prior fragility fractures [11]. This highlights the importance of considering recurrent fractures as an indicator of more severe osteoporosis and the need for comprehensive risk assessment.

Older women are particularly vulnerable, with fracture risk rising with advanced age combined with multiple clinical risk factors and additional low bone mineral density [7,8]. Osteoporosis prevalence varies between populations, influenced by lifestyle and genetics. For example, suburban Danish women have a higher osteoporosis prevalence than the Greenlandic population, and women are more affected than men [12].

### Fracture prevention

In addition to conventional pharmacological treatment for osteoporosis, several preventive strategies can reduce fracture risk. Individualized physical exercise programs, particularly weight-bearing and balance-focused training, have been shown to improve gait speed and muscle function, thereby reducing fall-related injuries and subsequent fractures [13–15]. Multifactorial interventions, such as home environment risk assessments, assistive devices (e.g. hip protectors and walking aids), and lifestyle adjustments, have also proven effective in reducing falls and fractures [13].

Nutritional supplementation with calcium and vitamin D has shown a modest reduction in hip and overall fracture risk among elderly women, although vitamin D alone has not demonstrated significant benefits [16].

Secondary prevention of fragility fractures has been shown to be cost-effective: in a Spanish study [17], the implementation of different actions to improve the prevention of secondary fractures produced a significant social benefit that, in terms of direct, indirect and intangible costs, far exceeded the investment.

Despite the availability of effective prevention strategies, many older individuals remain undertreated after sustaining a fracture. A Swedish population-based study of women aged 75 to 80 years found that only 21.8% of those eligible for osteoporosis treatment according to national guidelines received appropriate pharmacological therapy [18].

Thus, fragility fractures among older women remain common and have been associated with substantial personal, societal, and economic burdens. There is a clear need for effective, accessible, and sustainable prevention strategies within primary care settings.

### Aims

The aim of this non-randomized controlled intervention study was to evaluate the long-term impact of a primary care-based fracture prevention program on the incidence of fragility fractures among older Swedish women. The primary objective was to determine whether the intervention could reduce hip fracture incidence, with the original study powered to detect a 20% reduction of hip fractures after a 5-year follow-up period in the Vislanda women as compared to women in Emmaboda and Tingsryd.

A secondary objective was to investigate the incidence of fragility fractures and repeated fragility fractures during a 20-year follow-up period and their correlation with baseline characteristics, and intervention participation.

Additionally, the study aimed to assess the association between baseline risk factors, identified through a 22-item questionnaire completed in late 2001, and the risk of hip fracture.

## Materials and methods

### Study population

This population-based non-randomized controlled study initiated in 2001, presents a 20-year follow-up of 1,233 women aged 70–100 years who participated in a study focused on hip fracture prevention [19]. The study population, identified through the National Swedish Population Register, was drawn from women born between 1901 and 1931 residing in three rural communities in southeast Sweden: Vislanda, Tingsryd, and Emmaboda in 2001.

In Vislanda, all 501 eligible women were invited to participate in 2001, representing the entire female population within this age range in that community. These women formed the intervention group. For each Vislanda woman, an age-matched control was randomly selected from the female populations of Tingsryd and Emmaboda, two rural communities located 44 km and 94 km from Vislanda, respectively in southeast Sweden. Thus, for every Vislanda woman born between 1901 and 1931, one randomly selected woman of the same birth year was recruited from Tingsryd and another from Emmaboda, resulting in 997 women forming the control group. The control communities were demographically similar to Vislanda in terms of population structure, rurality, and access to healthcare services.

Of the 1,498 women invited to participate, 1,248 (83%) responded to the baseline questionnaire, including 435 women from Vislanda (87%), 418 from Tingsryd and 395 from Emmaboda (82%). By the follow-up in 2022, data from 1,233 women were analysed: 434 in the intervention group and 799 in the control group. One (1) woman in the intervention group and 14 women in the control group were lost to follow-up since they had moved away from the study area.

### Original questionnaire at baseline screening

In November 2001, a postal questionnaire was sent to all 1,498 women invited to participate in the study. The questionnaire consisted of 22 questions related to fracture risk, forming the basis of the baseline hip fracture risk screening database. Participants were allowed to receive help from family members or formal caregivers when completing the survey. The questions covered a broad range of areas, including: *Demographics*: age, weight, height, number of children. *Lifestyle factors:* diet, smoking, coffee intake, physical activity. *Medical history:* previous fragility fractures, number of regular medications used, mother's history of hip fracture, and menopause status. *Functional status:* recent falls, vision, and the ability to rise from a chair five times without using the arms. *Other factors:* self-rated health and general living conditions

### Interventions and follow-up (2002–2004)

Between 2002 and 2004, a graded fracture prevention program was conducted for 435 women in the intervention group (Vislanda) who responded to an extended baseline survey questionnaire of 38 items about knowledge of previous x-ray diagnosed vertebral fractures, current medication list, other chronic conditions etc. as well as consent for participation in intervention and fracture prevention follow-up (see original and translated versions of survey in appendix). At study onset, all 435 women in the intervention group (87%, 435 of 501) received the Walk Well Brochure (see original and translated versions of brochure in appendix) (Table 1).

**Table 1.** Study design for intervention and interim follow-up in three phases in a 20-year follow-up (2002–2021) of a population-based, non-randomized controlled fracture prevention study in southeast Sweden.

*Resources*	*Period*	*Persons*	*Contents*	*Tests/measures*
**Questionnaire respondents**	2001 Nov	435	Baseline questions, see Table 2.	Mobility & HF risk
**Interventions**				
Phase I:				
*Brochure Walk Well*	2002 Feb	435	Fall- & fracture preventive advice	–
Phase II:				
Home visit I by nurse*	2003 Feb	66	Life style advice, home training & fall prevention, drugs, hip pads, etc.	Mobility tests
Home visit II “*	2003 Apr	65		
*Group training by physiotherapist*	2003 Apr	28	Functional mobility training x 8. ‡ Fall prevention. Relaxation	Mobility tests
Phase III:				
*BMD + questionnaire*	2003 Oct	285	BMD heel. Mobility & fracture preventive advice.	Heel BMD DXL Mobility & hip fracture risk
Home visit III by nurse*	2004 Feb	62	Lifestyle advice, home training & fall prevention, drugs, hip pads, etc.	Mobility tests
*Physician follow-up of Home visits & low BMD*	2004 Feb	206	BMD heel and hip fracture risk questionnaire Home visits evaluated for lifestyle, physical training fall prevention, hip pads and drug use/treatments.	BMD & quest. Results home visit nurse report DXA hip & spine (n = 41)
**Questionnaire respondents**	2004 May	103	Questions on mobility, fracture etc.	Mobility & hip fracture risk
**Fracture registration**	2002–2021	434	Fractures in X-ray records	Hip and fragility fractures

*Home visits by nurse were offered to those having at least 2 clinical risk factors in Risk Model II: age ≥80 years, weight <60 kg, a previous fall within the past year, or a history of fragility fracture.

‡Group training offered for 8 sessions for 2 months periods; 28 women did 3–15 sessions.

### Phase I – universal intervention (2002) for the 435 women

Walk Well Brochure including general lifestyle advice promoting mobility, outdoor walking, and a healthy diet, guidance on fall and fracture prevention at home, home safety guidance, and written instructions for home-based exercises targeting balance and strength.

### Phase II – targeted high-risk intervention (2003–2004) for 66 women

Based on a four-factor risk model (age ≥80 years, weight <60 kg, a previous fall within the past year, or a history of fragility fracture), 103 women in the intervention group were identified as high risk (Risk Model 2 in [20]). Of these, 66 consented to participate in a one-year multifactorial, home-based intervention led by trained nurses. The program included three structured home visits at 0, 2, and 12 months, focusing on personalized fall risk assessment and support. As part of the intervention, group-based exercise sessions led by a physiotherapist at the primary care center were also offered, with 28 participants.

The intervention included: *Health education and exercise support*, such as personalized lifestyle advice (on walking, nutrition, and smoking cessation), written instructions for home exercises, a fridge magnet reminder, and re-distribution of the Walk Well Brochure.

*Assessment and provision of assistive aids*, including walking supports and hip protectors when indicated.

*Follow-up and evaluation,* including motivational support to enhance adherence and repeated mobility testing at each visit (timed chair rise test and balance assessment).

### Phase III: Bone mineral density screening and tailored interventions (late 2003–2004) for 285 women

In late 2003, all 390 women out of 435 women (90%) who were still alive were invited to complete a follow-up 15-item questionnaire and undergo a heel BMD DXL bilateral measurement [21]. A total of 285 out of 390 women (73%) participated. The main reasons for non-participation among the remaining 105 women included lack of interest, frailty, cognitive impairment, or logistical challenges.

Based on their BMD results and individual fracture risk profiles—including factors such as high age, previous fragility fracture, and low BMD—the women received tailored advice from physicians and physiotherapists.

#### Comparison of DXA and DXL measurements

41 women underwent both heel BMD assessment using the portable DXL technique and hip and spine BMD assessment using the standard DXA method. This included 30 consecutively selected participants and an additional 11 women who had heel T-scores ≤ −3.5 SD [21]. BMD predicted crude hip fracture risk by an odds ratio of 2.7 for every SD decrease in DXL T-Score and an odds ratio of 2.1 in an age adjusted model [21]. That study indicated that heel BMD measurements using DXL tended to yield lower T-scores compared to hip and spine measurements obtained *via* DXA. A heel T-score of −3.2 SD corresponded to a hip T-score of −2.5 SD, which was a suggested threshold for diagnosing osteoporosis [21].

All 285 participants received written information and advice by mail. Additionally, 206 of the women were contacted directly by a physician to discuss pharmacological treatment options based on their hip fracture risk. For women with a history of fragility fracture, calcium and vitamin D supplementation was considered at a heel T-score ≤ −2.0 SD, and bisphosphonate treatment was considered at a heel T-score of approximately ≤ −3.0 to −3.5 SD [21].

Individual exercise programs were also offered, often supervised by physiotherapists, as part of the tailored intervention strategy.

#### Pharmacological treatment uptake (2003–2004)

In October 2003 the 285 women who underwent BMD measurement, 42 (15%) were already taking vitamin D and 24 (8%) were on bisphosphonate therapy. By 2004, following the implementation of the tailored intervention program, 115 women (40%) were using vitamin D and 37 women (13%) were receiving bisphosphonate treatment.

### Control group

Women in the control group, residing in Tingsryd and Emmaboda, received usual care throughout the 20-year study period and received no intervention from us. Yet in the Tingsryd control area, some citizens over 80 years of age receiving municipal home care were offered a limited fall preventive community project from 2001.

### Fractures

Fracture data were retrospectively retrieved from radiology records, documenting fracture type, affected body side (right/left), and the date of occurrence. Fragility fractures were defined as those involving the hip (cervical, trochanteric, or subtrochanteric), wrist, proximal humerus, pelvic bone, and vertebral spine. Ankle fractures were also recorded but were considered less likely to be associated with fragility. All fractures occurring between January 1, 2002, and December 31, 2021, were registered based on the date of radiographic confirmation. Vertebral fractures were coded in the same way, acknowledging the uncertainty in determining the exact timing of the fracture relative to the date of the radiology report. Pelvic fractures were mostly stable fractures located in the superior and inferior pubic rami, but we did not group them by specific location or side.

Baseline variables were obtained from the 22-item questionnaire administered in the original study in 2001. Variables included age at study start, previous fragility fracture (after age 50), age ≥80 years, weight <60 kg, ability to rise five times from a chair without arm support, history of a fall in the past year, maternal history of hip fracture, height >167 cm, self-reported poor health, impaired vision, nulliparity, cortisone treatment for >3 months, current smoking, daily coffee intake (≥2 cups), daily calcium intake <500 mg, menopausal age <45 years, and study group allocation (intervention or control).

### Ethical considerations

Baseline data were retrieved from the original 2001 study, while additional fracture and mortality data were collected through a review of patient medical records from 2002 to 2021. All women participated voluntarily and provided written informed consent when responding to the original questionnaire.

Ethical approval for this follow-up study was granted by the Swedish Ethical Review Authority (Dnr 2021–03866, August 30, 2021), while the original study was approved by the Regional Ethics Committee at Lund University (LU 406-00, LI 00–218, January 8, 2001). This trial was registered at ClinicalTrials.gov (Identifier: NCT05269979) before study initiation.

### Statistical methods

Statistical analyses were performed using SPSS version 28. A two-sided p-value <0.05 was considered statistically significant.

The original study was powered to detect a 20% reduction in hip fracture incidence between the intervention and control groups after a 5-year follow-up period. Based on an anticipated hip fracture incidence of 10% over three years in the control group, a sample size of 1,200 women (400 in the intervention group and 800 in the control group) was estimated to provide 80% power to detect a 20% difference, with a two-sided significance level of 0.05. The 20-year follow-up extended this evaluation to assess long-term intervention effects.

Comparisons between the intervention and control groups were conducted for different fracture types and their associations with baseline risk factors. The following statistical methods were applied: Fisher's exact test for categorical variables. Multiple Cox regression models were applied to estimate hazard ratios (HR) and 95% confidence intervals (CI) for hip and repeated fragility fractures, adjusting for baseline risk factors. Multiple logistic regression models were used to adjust for baseline risk factors for the outcome repeated fragility fractures.

Fracture incidence rates were calculated per 1,000 person-years and presented in five-year age categories. These rates were derived by dividing the number of fractures at each site within an age group by the total number of person-years lived by women in that age group.

Vertebral fractures were analyzed as prevalence and not incident data due to uncertainty regarding precise fracture dates.

Missing data were handled using a conservative approach: non-responses were interpreted as indicating low risk in order to avoid overestimating the potential risk of hip fracture in the analysis. This classification allowed us to retain as much data as possible from all participants in the multiple regression models. Three baseline variables had internal response rates between 86% and 93%, while the remaining variables had response rates of 95% to 100%.

Proxy responses were permitted, with relatives or caregivers assisting participants in completing the baseline questionnaire when needed. The study design facilitated inclusion of high-risk participants by ensuring the questionnaire consisted of simple, clear, and concise questions to improve response accuracy and reduce missing data.

The analysis was conducted without blinding, as researchers were aware of participants' group allocation (intervention or control).

This study adhered to the Transparent Reporting of Evaluations with Nonrandomized Designs (TREND) checklist to ensure comprehensive reporting of nonrandomized controlled intervention studies [22], see Appendix.

## Results

Recruitment for the study took place between November 2001 and January 2002. Invitations were sent to all eligible women born between 1901 and 1931 in Vislanda, Tingsryd, and Emmaboda, accompanied by a motivational cover letter explaining the study's purpose. Two reminders were sent to non-respondents, and the majority (>90%) of responding participants were recruited in late 2001.

**Figure 1.**
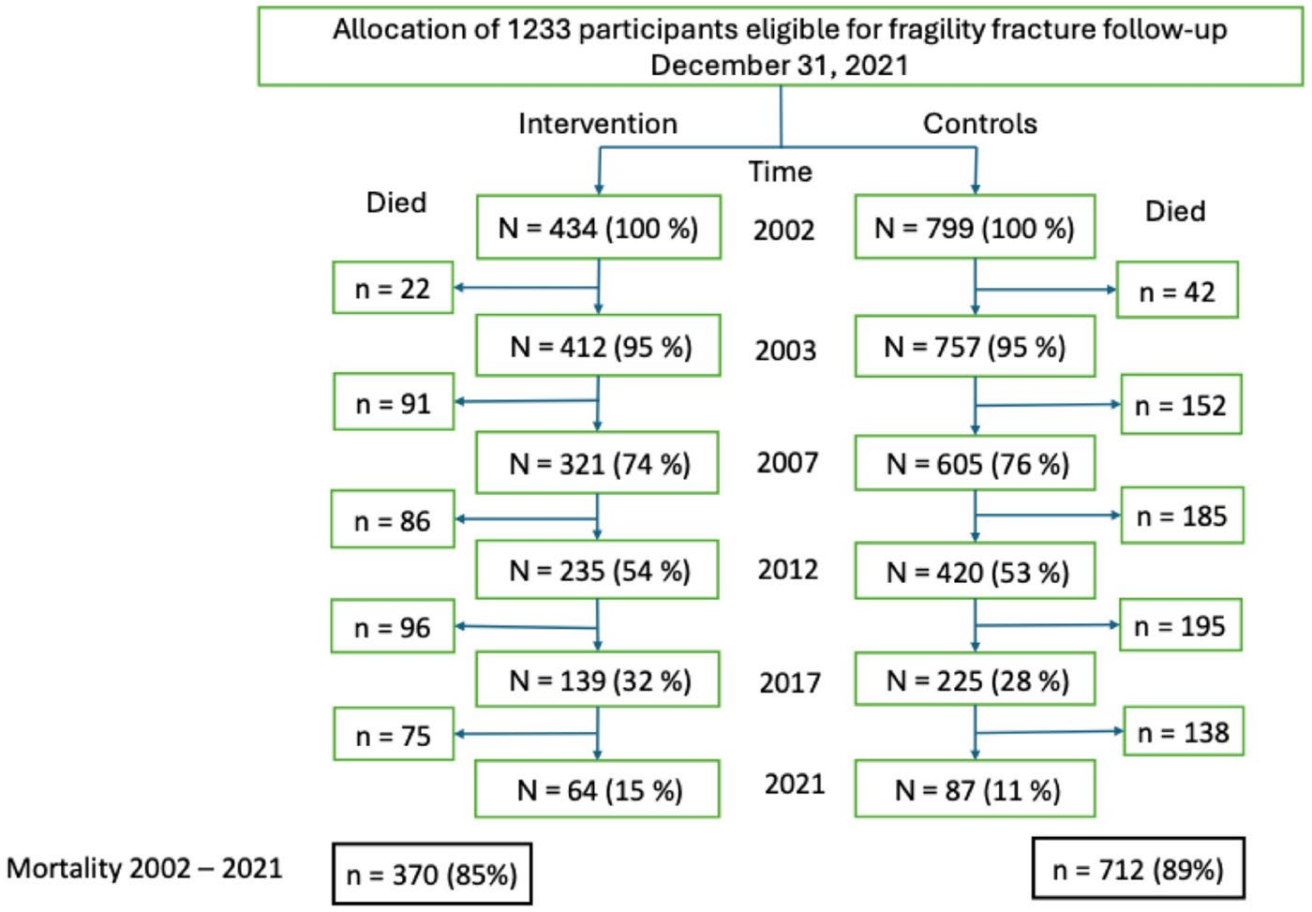
Study population mortality flowchart. 1,233 women at the end of 2021 were included in this 20-year follow-up study (n = 434 intervention group, n = 799 control group). At follow-up, 1,082 women had died, 370 in intervention group, 712 in control group. Participants lost to follow-up (n = 15) had relocated outside the study areas.

**Figure 2.**
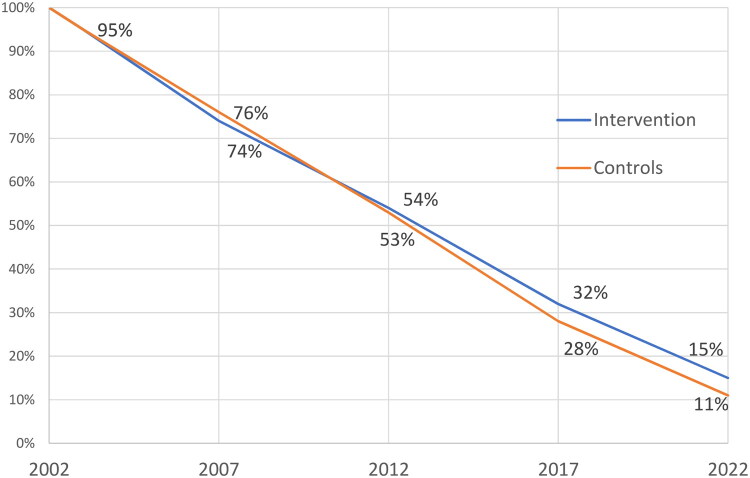
Study population survival proportion curves. Kaplan–Meier analysis indicated a borderline significant difference (log-rank p = 0.056), with an estimated hazard ratio of 0.86 (95% CI ∼0.73–1.00). 1,233 women at the end of 2021 were included in this 20-year follow-up study. At follow-up 2021, 151 women (12%) were alive, 64 (15%) in intervention group and 87 (11%) in control group. Participants lost to follow-up (n = 15) had relocated outside the study areas.

This 20-year follow-up study included 1,233 women aged 70 years and older at baseline, with a mean age of 79.4 years. By the end of the study period in 2021, 1,082 women (88%) had died, while 151 women (12%) were still alive, with a mean age of 93.9 years. The proportion of surviving women was 64/434 (14.7%) in the intervention group compared to 87/799 (10.9%) in the control group (*p* = 0.056), see Figures 1 and 2.

Baseline characteristics of the intervention and control groups are shown in Table 2. Women in the control group were more likely to report falls within the past 12 months (*p* = 0.002), subjectively poorer health (*p* < 0.001), and lower calcium intake (*p* = 0.011) compared to the intervention group. Cortisone medication for more than three months was more frequent in the control group, though the difference was not statistically significant (*p* = 0.053).

**Table 2.** Baseline caracteristics of the intervention and control group, retrieved from the baseline questionnare completed by women aged > 70 years in 2001 (*n* = 1233).

	Total	Intervention	Control	
	(*n* = 1233)	(*n* = 434)	(*n* = 799)	
	n (%)* or	n (%)* or	n (%)* or	
Characteristics	mean**	mean**	mean**	p value‡
***Risk model risk factors***				
Age in 2001	79.4 ± 6,4	79.5 ± 6.6	79.3 ± 6.4	0.650
Age > 80 years	490(39.7)	176(40.6)	314(39.3)	0.670
Weight < 60 kg	301(24.4)	105(24.2)	196(24.5)	0.945
Need arms when rising from chair 5 times	361(29.3)	124(28.6)	237(29.7)	0.695
Fall during last 12 months	392(31.8)	114(26.3)	278(34.8)	0.002
Previous fragility fracture > age 50	375(30.4)	125(28.8)	250(31.3)	0.400
***Other possible risk factors***				
History of maternal hip fracture	121(9.8)	40(9.2)	81(10.1)	0.689
Height > 167 cm	179(14.5)	63(12.4)	116(15.6)	0.150
Subjective poor health	118(9.6)	25(5.8)	93(11.6)	<0.001
Impaired vision (self-reported)	355(28.8)	114(26.3)	241(30.2)	0.167
Has never given birth	161(13.1)	62(14.3)	99(12.4)	0.376
Cortisone medication > 3 months	147(11.9)	41(9.4)	106(13.3)	0.053
Current smoking in 2001	50(4.1)	16(3.7)	34(4.3)	0.763
Daily coffee intake ≥ 2 cups	1026(83.2)	356(82.0)	670(83.9)	0.426
Dairy calcium intake (< 500 mg/d)	371(30.1)	111(25.6)	260(32.5)	0.011
Menopausal age < 45 years	126(10.2)	37(8.5)	89(11.1)	0.168

*Estimated percentage of participants.

**Mean value is given as arithmetic mean ± SD.

‡Exact p value according to the Fisher's Exact Test, 2-sided for dichotomous factors and estimated p value according to logistic regression for continous factors.

At the 20-year follow-up in January 2022, a total of 151 women (12.3%) were still alive. The proportion of surviving women was higher in the intervention.

group (64/434, 14.7%) compared to the control group (87/799, 10.9%), *p* = 0.053, Chi-Square test.

### Fractures

Between 2002 and 2021, 236 women (19%) sustained 268 hip fractures, making it the most common fragility fracture, and 32 women (2.6%) sustained two hip fractures. Hip fractures occurred in 17.7% of women in the intervention group (77/434) compared to 19.9% in the control group (159/799), representing an 11% lower relative incidence in the intervention group. This difference was not statistically significant (*p* = 0.36), and the study's original aim of achieving a 20% reduction in hip fractures within the intervention group was not reached.

During the 20-year follow-up, 884 fractures were identified among 536 women (43% of the cohort), corresponding to an overall incidence rate of 679 fractures per 10,000 person-years, including prevalent vertebral fractures (Table 3).

**Table 3.** Fracture site, total number of fractures, and total number of unique women with fracture, in elderly women aged >70 years old (*n* = 1233), in Southern Sweden 2002–2021.

Fracture site	Fractures n* (%)**	Unique women with fracture n (%)§
All fractures	884	536(43.5)
Hip	268(30.0)	236(19.1)
Vertebra	233(26.0)	183(14.8)
Wrist	162(18.0)	148(12.0)
Pelvis	104(12.0)	100(8.1)
Proximal Humerus	89(10.0)	84(6.8)
Ankle	28(3.0)	28(2.3)

*Fractures of the hip, wrist, pelvis, proximal humerus and ankle fractures are presented as incident fractures whereas vertebral fractures are presented as prevalent fractures.

**Percentage of all fractures (*n* = 884).

§Percentage of all women (*n* = 1233).

A total of 233 prevalent vertebral fractures were registered in 183 women (15%). Wrist fractures were the third most common fracture site, with 162 fractures in 148 women (12%), of which 72 (44%) affected the right arm and 90 (56%) the left. Additionally, 104 pelvic fractures in 100 women (8%) and 89 proximal humerus fractures in 84 women (7%) were recorded, with no significant side difference.

### Fragility fracture distribution

The Fragility Fracture 5 (FF-5) index was used to assess each woman's cumulative number of fragility fractures occurring in the hip, wrist, proximal humerus, vertebrae, and pelvis between 2002 and 2021. Ankle fractures were excluded due to uncertainty regarding their relationship to fragility.

A total of 210 women (17%) sustained two or more FF-5 fractures (Table 4). Significantly fewer women in the intervention group (14.1%) than in the control group (18.6%) had two or more fragility fractures during the study period (odds ratio (OR) 0.71; 95% CI, 0.53–1.0; *p* = 0.047). This difference remained significant after regression analysis adjustment including the four baseline factors differing between the groups with *p* < 0.15 shown in Table 2.

**Table 4.** Classification of 1233 women aged >70 years by fragility fracture 5 (FF-5) index: distribution of participants with 0–1 fracture versus those with ≥2 fragility fractures in a 20-year follow-up (2002–2021) of a population-based, non-randomized controlled fracture prevention study in southeast Sweden.

	Fractures n (%)*				
Number of fractures	Total	Intervention group	Control group	OR (95% CI)	p value‡
	*n* = 1233	*n* = 434	*n* = 799		
0–1	1023 (83.0)	373 (85.9)	650 (81.4)	–	–
≥2	210 (17.0)	61 (14.1)	149 (18.6)	0.71 (0.53–1.0)	.047

OR = odds ratio; CI = confidence interval.

*Percentage of women.

‡Exact P value according to the Fisher's Exact Test.

*''*Fragility Fracture 5 (FF5) consisting of added fractures of the hip, wrist, proximal humerus, vertebrae, and pelvis.

Among 236/1233 women who had sustained hip fracture 32 out of 77 women (42%) in the intervention group had also sustained at least one other fragility fracture whereas 90 out of 159 women (57%) in the control group who had sustained a hip fracture also had at least one other fragility fracture (OR 0.54 (95% CI 0.31–0.95), *p* = 0.037).

Among the 997/1233 women who had not sustained a hip fracture 29 out of 357 women (8.1%) in the intervention group had sustained two or more fragility fractures and 59 out of 640 women (9.2%) in the control group had sustained two or more fragility fractures (OR 0.87 (95% CI 0.55–1.39) *p* = 0.64).

### Fracture incidence rates

**Figure 3.**
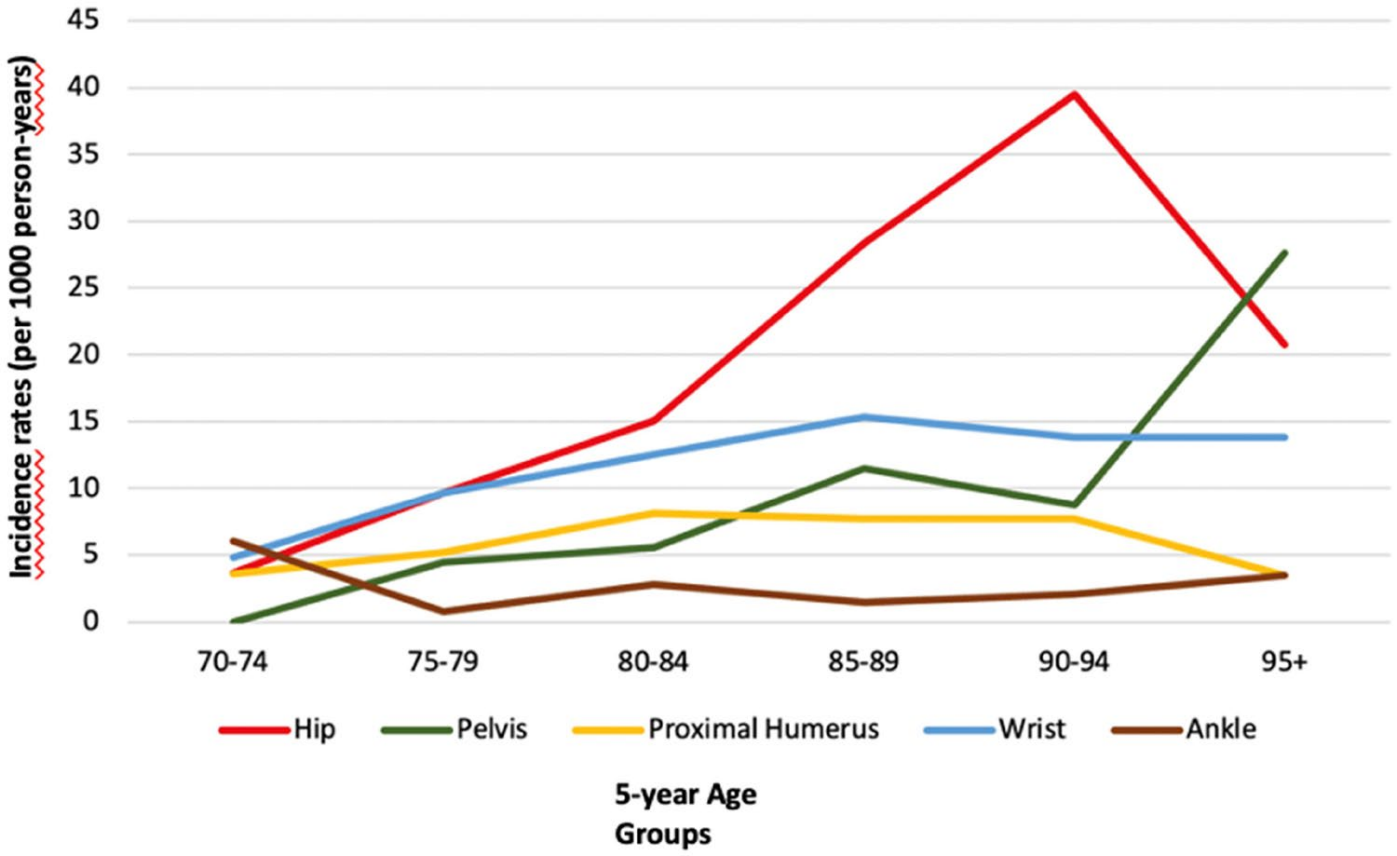
Fracture incidence rates (per 1,000 person-years) by fracture site and 5-year age groups in 1233 women aged >70 years in a 20-year follow-up (2002–2021) of a population-based, non-randomized controlled fracture prevention study in southeast Sweden.

The 20-year fracture incidence rates per 1,000 person-years for each fracture site, stratified by five-year age groups, are presented in Table 5 and Figure 3 (vertebral fractures were not counted as incident fractures due to uncertainty regarding precise fracture dates).

**Table 5.** 20-Year fracture incidence (per 1000 person-years) in elderly women aged > 70 years old (*n* = 1233), in Southern Sweden 2002–2021, divided into 5-year age groups.

	Age groups (n)*						
	70–74	75–79	80–84	85–89	90–94	95+	
	(*n* = 355)	(*n* = 728)	(*n* = 911)	(*n* = 879)	(*n* = 663)	(*n* = 240)	
Person-years**	827.6	2700.0	3583.9	3386.2	1950.1	579.3	
Fracture site		Number of fractures per 1000 person-years n					n§
Hip	3.6	9.6	15.1	28.4	39.5	20.7	268
Wrist	4.8	9.6	12.6	15.4	13.8	13.8	162
Proximal Humerus	3.6	5.2	8.1	7.7	7.7	3.5	104
Pelvis	0.0	4.4	5.6	11.5	8.7	27.6	89
Ankle	6.0	0.7	2.8	1.5	2.1	3.5	28

*Number of women that were ever alive in the age group.

**Total number of person-years in the age group.

§Total number of fractures for each fracture site.

Hip fractures had the highest overall incidence rate, with 39.5 fractures per 1,000 person-years among women aged 90–94 years, with consistently high rates (>20 per 1,000 person-years) among women aged 85 years and older.

Wrist fractures peaked in the 85–89 age group, with 15.4 fractures per 1,000 person-years, while proximal humerus fractures were most common in the 80–84 age group, with an incidence rate of 8.1 per 1,000 person-years. Pelvic fractures had the highest incidence in women aged 95 years and older, with 27.6 fractures per 1,000 person-years.

Among all incident fractures, 79.3% occurred in women aged 80 to 94 years. The mean age at the first hip fracture was 87.0 years, while for first wrist fracture mean age was 85.6 years.

Hip fractures were associated with the highest one-year mortality rate (27%), followed by pelvic fractures (26%). The mean survival time after a first hip fracture was 2.9 years.

### 20-Year hip fracture risk

Table 6 shows the 20-year risk of hip fracture in relation to baseline characteristics and risk factors. The multivariable Cox regression model included 16 baseline factors. Age emerged as the strongest predictor of hip fracture: compared to women aged 70–74 years at baseline, the hazard ratio (HR) was 1.8 (95% CI: 1.3–2.5) for those aged 75–79 years, 2.8 (1.9–4.1) for ages 80–84, and 4.0 (2.6–6.2) for women aged 85 years and older.

**Table 6.** Twenty-year hip fracture risk by clinical risk factors in 1233 women aged >70 years in a 20-year follow-up (2002–2021) of a population-based, non-randomized controlled fracture prevention study in southeast Sweden.

	First Hip	No Hip		Multivariate^‡^	Multivariate^‡^
	Fracture	Fracture	*P* ^†^	4 Factors	16 Factors
Characteristics	= 236 (%)	= 997 (%)	Exact	HR (95% CI)	HR (95% CI)
**Age categories** ^§^					
Age 70–74	58(24.6)	297(29.8)	0.039	Reference	Reference
Age 75–79	88(37.3)	300(30.1)		1.7 (1.2–2.4)	1.8 (1.3–2.5)
Age 80–84	51(21.6)	184(18.5)		2.6 (1.8–3.9)	2.8 (1.9–4.1)
Age 85+	39(16.5)	216(21.7)		3.4 (2.1–5.3)	4.0 (2.6–6.2)
***Intervention vs Control***					
Intervention group^#^	77(32.6)	357(35.8)	0.364	–	N.S
***Risk model risk factors***					
Weight < 60 kg	61(25.8)	240(24.1)	0.557	1.4 (1.0–1.9)	1.5 (1.1–2.0)
Need arms when rising from a chair 5 times	54(22.9)	307(30.8)	0.017	1.4 (1.0–2.0)	1.4 (0.98–1.9)’’
Previous fragility fracture > age 50^¶^	80(33.9)	295(29.6)	0.208	1.3 (0.97–1.7)’’	1.3 (0.96–1.7)’’
Fall during last 12 months	74(31.4)	318(31.9)	0.938	–	N.S
***Other possible risk factors***					
History of maternal hip fracture	30(12.7)	91(9.1)	0.113	–	N.S
Height > 167 cm	44(18.6)	135(13.5)	0.051	–	1.6 (1.1–2.2)
Subjective poor health	14(5.9)	104(10.4)	0.036	–	N.S
Impaired vision (self-reported)	68(28.8)	287(28.8)	1.000	–	N.S
Has never given birth	24(10.2)	137(13.7)	0.163	–	N.S
Cortison medication > 3 months	33(14.0)	114(11.4)	0.266	–	N.S
Current smoking in 2001	9(3.8)	41(4.1)	1.000	–	N.S
Daily coffee intake ≥ 2 cups	209(88.6)	817(81.9)	0.015	–	1.4 (0.95–2.1)’’
Dairy calcium intake (<500 mg/d)	76(32.2)	295(29.6)	0.431	–	N.S
Menopausal age < 45 years	26(11.0)	100(10.0)	0.634	–	N.S

HR = hazard ratio; CI = confidence interval; N.S = Not significant.

*Estimated percentage of women with first hip fracture and those with no hip fracture (during the study period).

^†^Exact *P* value according to the Fisher exact test, 2-sided.

^‡^Cox regression analysis.

^§^Age categories divided according to the women's age at the start of the study in 2002.

^#^Belongs to intervention group vs control group.

^¶^Previous fragility fracture in the hip, lower arm, upper arm, or vertebrae ≥ 50 years.

**p <0.05.

‘‘0.05 > *p* < 0.10.

#Reported vertebral fractures included from responses to the question about radiographs posed only to the Vislanda population.

§The variable “any type of fracture” was excluded from multivariate analysis because it competed with and was highly correlated with “previous fragility fracture.” ǁ *p* <0.05.

Other significant predictors included low body weight (<60 kg; HR = 1.5, 95% CI: 1.1–2.0) and tall stature (>167 cm; HR = 1.6, 95% CI: 1.1–2.2). Additional factors such as the need for arm support when rising from a chair, prior fragility fractures, and daily coffee intake showed borderline associations with hip fracture risk.

Group assignment (intervention vs control) was not significantly associated with hip fracture risk (HR = 0.89, 95% CI: 0.70–1.14; marked as NS in Table 6).

## Discussion

This 20-year follow-up study examined bone fractures among 1,233 rural Swedish women with baseline ages of 70–100 years who participated in a nonrandomized controlled intervention aimed at preventing hip fractures, initiated in 2001 [19].

The peak number of fractures occurred among women aged 85 to 89 years, with nearly 80% of all incident fractures observed in women aged 80 to 94 years. Hip fracture was the most common fragility fracture, with the highest incidence rate in women aged 90 to 94 years. Pelvic fractures had the highest incidence among women aged 95 years and older and were associated with the second highest mortality rate after hip fractures.

The intervention aimed to reduce hip fracture incidence through fall prevention strategies, physical activity promotion, home-based safety adjustments, and pharmacological treatment when indicated. The primary objective of the study was to achieve a 20% reduction in hip fractures among women in the intervention group compared to the control group. While the intervention group experienced an 11% lower relative incidence of hip fractures this difference was not statistically significant. The inability to achieve the targeted reduction may be attributed to the limited duration and intensity of the intervention.

The risk of sustaining two or more fragility fractures during the study period was significantly lower in the intervention group than in the control group. Notably, the combined outcome of a hip fracture and at least one more additional fragility fracture occurred even less frequently in the intervention group. The intervention was thus associated with a reduced cumulative fracture burden among high-risk individuals.

Regression analyses identified three significant baseline risk factors for hip fracture: advanced age, body weight under 60 kg, and height over 167 cm which remained significant after adjustment for potential confounders. These risk factors have been described previously [7,9].

### Comparison with other studies

The mean age of women sustaining a hip fracture during the study was 87 years. While this is four years older than the average life expectancy for Swedish women at birth it aligns with the remaining life expectancy for Swedish women aged 80, which was approximately nine years in 2002 [23] and the study cohort's average age at baseline 2001 was 79.4 years.

The highest hip fracture incidence rate in the current study was observed among women aged 90 to 94 years, with 39.5 fractures per 1,000 person-years. In contrast, Ref. [5] reported the highest incidence among Swedish women aged 95 years and older, with approximately 50 hip fractures per 1,000 person-years. This discrepancy may be explained by our inclusion of women with prior hip fractures at study start, leading to an overestimation of person-years at risk and an underestimation of incidence rates in the oldest age groups.

The mean age for wrist fractures in this study was 85.6 years, reflecting the inclusion of women aged 70 years and older, many of whom had already experienced wrist fractures prior to the study. Ref. [24] found that most wrist fractures in Swedish women occurred between the ages of 60 and 69 years, with a mean age of 65.4 years.

Notably, in our study we observed 27% more left hand wrist fractures than right hand wrist fractures consistent with previous findings [24]. The higher incidence of left wrist fractures may be attributed to a combination of behavioral factors, such as the tendency to use the non-dominant hand for protection during falls [25], and physiological factors, including lower bone density and muscle strength in the non-dominant limb [26].

### Strengths and limitations

The study's strengths include its population-based design, high response rate, and long-term follow-up. The original questionnaire in 2001 had an overall response rate of 83%, with individual questions typically achieving response rates above 95% [19]. Missing responses were conservatively coded as indicating low risk to avoid overestimating risk associations, though this approach may have led to underestimation of certain risk relationships.

Fracture outcomes were based on radiology reports 2002–2021, providing accurate information regarding fracture site, laterality, and date of the incident. However, prevalent vertebral fractures were often detected incidentally, and their exact occurrence dates were uncertain, preventing us to include them in incidence rate calculations.

Another limitation was the lack of blinding during data analysis. Researchers were aware of participants' allocation to either the intervention or control group, potentially introducing bias in outcome interpretation. Although fracture diagnoses were based on objective radiology reports, awareness of group assignment could have influenced data handling and interpretation.

Another limitation of this study is that fracture data were only collected from radiology departments at hospitals in Kalmar and Kronoberg regions. Fractures that occurred while participants were away from home and treated at hospitals in other regions were not recorded, leading to an underestimation of total fracture incidence. Also, there are reports indicating that some fractures are diagnosed uniquely in primary care, albeit mostly wrist fractures and sport injuries in younger people and in remote areas [27,28].

The study's generalizability is limited by its focus on a rural Swedish population, which may differ from urban or international populations in terms of health status, lifestyle, and healthcare access. Additionally, the inclusion of women aged on average 79.4 years at baseline means that incidence rates cannot be extrapolated to younger old populations.

The nonrandomized design increased the risk of selection bias. Although the control group was age-matched and drawn from similar rural areas (Tingsryd and Emmaboda), unmeasured confounding cannot be excluded.

One important limitation is the observed baseline imbalance in subjective poor health between the intervention and control groups. A significantly higher proportion of women in the control group reported poor self-rated health at baseline (11.6% vs. 5.8%, *p* < 0.001). Since subjective health has been associated with fracture incidence [29], this difference may have contributed to the observed reduction in repeated fragility fractures in the intervention group. However, the difference remained statistically significant after adjustment for baseline variables that significantly differed between the groups including subjective poor health. This implies that the findings of reduced fragility fractures in the intervention group were not explained by baseline imbalance.

At the 20-year follow-up, a slightly higher proportion of women in the intervention group were still alive. Yet, this difference was not significant and our study was not designed to examine longevity or mortality differences.

In the Tingsryd control area, a limited fall prevention initiative was introduced in 2001 to some home care recipients over the age of 80. This may have partly diluted the observed possible effects of the intervention.

### Implications for practice and future research

This long-term follow-up population based study provides valuable insights into the epidemiology of fragility fractures among old women, underscoring the persistent risk associated with older age, low body weight, and taller stature – findings in this study that confirm previously known risk factors [7,9]. This highlight the importance of early identification and targeted preventive strategies for high-risk individuals.

The lower proportion of repeated fragility fractures in the intervention group—particularly among those who had sustained a hip fracture—may suggest that certain fracture types are more responsive to early intervention. This observation could also be influenced by the elevated short-term risk following an initial fracture and the limited statistical power to detect differences in hip fracture incidence alone.

Multifactorial interventions as in our study - including physical exercise, home safety modifications, and pharmacological treatment - have been shown to reduce fall-related injuries and fractures in older adults [13,15]. Furthermore, the Social Return on Investment (SROI) for preventive measures targeting fragility fractures in postmenopausal women with osteoporosis has been high in studies from Spain [17,30].

Future studies could include repeated measurements of risk factors, recognizing that variables such as recent falls, ability to rise from a chair, cortisone use, and self-rated health are dynamic and may change over time. The importance of reassessing risk is further emphasized by evidence that fracture risk increases significantly following an initial fragility fracture, particularly within the first two years [2]. Comprehensive assessments of bone mineral density (BMD), including both baseline and follow-up measurements across clinical risk groups, would allow for more accurate and updated risk stratification [7,31].

Further research should explore whether graded hip fracture risk prevention interventions with prolonged or repeated exercise periods combined with pharmacological treatment can offer sustained fracture prevention. Randomized controlled trials have shown that supervised, weight-bearing exercise can improve muscle strength, balance, and gait speed, thereby reducing the risk of falls and subsequent fractures [14,32]. Longitudinal studies with larger cohorts and repeated risk assessments could offer a more nuanced understanding of how changing health status influences fracture risk over time.

The long-term effects of our intervention - fall and fracture prevention guidance, written home exercise instructions, hip fracture risk assessment, and treatment suggestions - may have enhanced empowerment and daily activity among participants. A smaller high risk group also received follow-up and motivational support from nurses. We suggest that this type of graded fracture prevention program be repeated, with several interim follow-ups within existing primary care frameworks. Also, a formal health economic evaluation is warranted to assess its potential cost-effectiveness.

## Conclusions

This 20-year follow-up study of a non-randomized controlled primary care intervention aimed at preventing hip fractures in 1,233 rural Swedish women aged 70–100 years at baseline found a non-significant reduction in hip fracture incidence in the intervention group compared to the control group. However, the intervention was associated with a significantly lower incidence of recurrent fragility fractures, especially for women having suffered hip fractures. These findings suggest that a graded, primary care, home and community-based intervention - focused on fall prevention incorporating guided physical training and bone-strengthening treatment for individuals at high risk of hip fracture - may help reduce the risk of subsequent fractures.

## Supplementary Material

TREND checklist for appendix.docx

HIP22_survey_English_Extended_Intervention_250619.pdf

walk_well_original_2015.pdf

Figure 1A.pptx

Survey_HIP22_baseline_Swedish.pdf

Walk Well BROCHURE.docx
