# Supplementary material for: Reduced Risk of Recurrent Fragility Fractures After a Primary Care–Based Fracture Prevention Intervention: A 20-Year Non-Randomized Controlled Follow-Up Study in Women Aged 70–100
Source: Scand J Prim Health Care. 2025 Nov 6;44(1):1–16. doi: 10.1080/02813432.2025.2571929 (PMC12918357; doi:10.1080/02813432.2025.2571929)
Supplement: TREND checklist for appendix.docx [file IPRI_A_2571929_SM4452.docx]

**Appendix: TREND Checklist**

**Transparent Reporting of Evaluations with Nonrandomized Designs (TREND)**
Reference: Des Jarlais et al., *Am J Public Health* 2004;94:361–366.
This checklist shows how each item has been addressed in the manuscript:

| **Section** | **Item** | **Descriptor** | **Location in Manuscript** |
| --- | --- | --- | --- |
| **Title and Abstract** | 1 | Allocation method, intervention, and study design clearly stated | Title and Abstract (page 1) |
| **Introduction** | 2 | Background and rationale | Introduction (pages 2–4) |
| **Objectives** | 3 | Specific objectives or hypotheses | Aims (page 5) |
| **Methods: Participants** | 4 | Eligibility criteria, settings, locations, how participants were selected | Study Population (pages 6–7) |
| **Methods: Interventions** | 5 | Description of interventions with sufficient detail | Interventions and Follow-Up (pages 7–9) |
| **Methods: Objectives** | 6 | Primary and secondary outcome measures defined | Aims and Outcome Measures (page 5) |
| **Methods: Outcomes** | 7 | How and when outcomes were assessed | Fracture data, BMD assessment (pages 10–11) |
| **Methods: Sample size** | 8 | How sample size was determined | Statistical Methods (page 16) |
| **Methods: Assignment Method** | 9 | Method of assigning participants to groups | Nonrandomized allocation described (page 6) |
| **Methods: Blinding** | 10 | Blinding of participants, providers, assessors | Not blinded (page 16) |
| **Methods: Unit of Analysis** | 11 | Unit of analysis stated and consistent | Individual-level (pages 6, 16) |
| **Methods: Statistical Methods** | 12 | Methods used to compare groups, handle missing data, etc. | Statistical Methods (pages 16–17) |
| **Results: Participant Flow** | 13 | Flow of participants through each stage, including losses | Figure 1A and 1B (page 12) |
| **Results: Recruitment** | 14 | Recruitment and follow-up dates | Recruitment (page 12) |
| **Results: Baseline Data** | 15 | Demographic and baseline characteristics | Table 2 (page 13) |
| **Results: Numbers Analyzed** | 16 | Number of participants analyzed in each group | Results section (pages 12–14) |
| **Results: Outcomes and Estimation** | 17 | Summary of results and estimated effect sizes | Tables 3–6, Results (pages 13–15) |
| **Results: Ancillary Analyses** | 18 | Subgroup and adjusted analyses | Regression analyses (pages 15–16) |
| **Results: Adverse Events** | 19 | Adverse events or unintended effects reported | Not applicable (no adverse events) |
| **Discussion: Interpretation** | 20 | Interpretation of results, including limitations | Discussion (pages 18–22) |
| **Discussion: Generalizability** | 21 | Generalizability of findings to other settings/populations | Discussion: Strengths and Limitations (page 20) |
| **Discussion: Overall Evidence** | 22 | Summary of findings and conclusions | Conclusions (pages 22–23) |
