# Supplementary material for: Reduced Risk of Recurrent Fragility Fractures After a Primary Care–Based Fracture Prevention Intervention: A 20-Year Non-Randomized Controlled Follow-Up Study in Women Aged 70–100
Source: Scand J Prim Health Care. 2025 Nov 6;44(1):1–16. doi: 10.1080/02813432.2025.2571929 (PMC12918357; doi:10.1080/02813432.2025.2571929)
Supplement: HIP22_survey_English_Extended_Intervention_250619.pdf [file IPRI_A_2571929_SM4451.pdf]

HIP22 Questionnaire (English Translation) - Extended for intervention area

Do You Have a Risk of Hip Fracture?
Date: 2001-09-23

Thank you for participating in the previous survey about osteoporosis and falls. We hope you found the advice useful. The study will continue to be evaluated for several more years, and we will share more results later on.

Due to great interest, we have decided to send out a new questionnaire to all women over 70 in the Vislanda area. Your participation in this new survey would be greatly appreciated.

Osteoporosis is a disease that causes decreased bone density and an increased risk of bone fractures. The condition is becoming increasingly common—today, a woman has twice the risk of a fracture compared to the 1950s. The most common fractures occur in the forearm, spine, and femoral neck (hip fracture).

For many people, good everyday habits are enough to reduce the risk of fractures. For example, daily walking strengthens muscles and bones and improves balance. You can also reduce your fall risk by making your home environment safer or using anti-slip protection. For those at high risk of falling, hip protectors are available. Some people may also need calcium supplements in their diet or other medications to strengthen bones.

The purpose of this survey is to assess the risk of osteoporosis and falls among older women in the Vislanda area.

You are one of the women over 70 selected to participate.

If you choose to participate, we ask you to fill in the attached questionnaire and return it to us in the enclosed reply envelope. If you need help filling it out, feel free to ask a relative or home care staff for assistance.

Once we have received your form, we will send you a brief summary with advice. We will also contact anyone we believe might benefit from special treatment or further guidance. On page 5 (question 37) of the questionnaire, you can indicate whether you would like to be contacted by a nurse in that case.

The information from the questionnaire will be processed and handled in accordance with healthcare confidentiality laws.

Your participation is completely voluntary, and you can withdraw at any time without it affecting any other care you receive.

By participating in the study, you may reduce your risk of falling and sustaining a fracture.

Your answers are valuable to the study, even if you are currently in good health.

If you have any questions, please feel free to call a nurse (see contact info below) or **Daniel Albertsson** at the Vislanda Health Center (0472/30154).

Kind regards,
Daniel Albertsson, Project Manager, Physician
Roland Johansson, Head of Clinic, Physician
Mats Henriksson, Physician
Eva Hjerpe, Physician
Agneta Lönn, Nurse, Vislanda Health Center
Annika Magnusson, Nurse, Björkliden
Maria Lindahl, Nurse, Asken & Torsgården

Questions for Women Over 70 About Osteoporosis and Falls

Mark with a cross in one of the boxes for each question – unless otherwise stated.
Choose the box that suits you best, even if it's not entirely accurate.
You don't need to worry about the small reference numbers (1, 2, 3) in the form.
If you are unsure or want to add anything, feel free to write beside the question or call us.

---

1. How old are you now?
I am ___ years old.

2. How are you feeling these days?
☐ Excellent
☐ Fairly good
☐ Poor

3. Has your mother had a hip fracture (fracture of the femoral neck)?
☐ Yes (She was about ___ years old then)
☐ No
☐ Don't know

4. Have any of your parents or siblings had a fracture in the forearm, upper arm, spine, or hip during adulthood?
☐ Yes

☐ No
☐ Don’t know

5. How much milk, yogurt, or sour milk do you consume daily?
I drink ___ glasses of milk or bowls of yogurt per day.

6. How many cups of coffee do you drink per day?
I drink about ___ cups of coffee per day.

7. How many slices of cheese do you eat daily?
I eat about ___ slices of cheese per day.

8. Do you currently smoke?
☐ Yes
☐ No, but I used to
☐ No, I’ve never smoked → skip to question 10

9. How much have you smoked?
I started smoking at age ___ and quit ___ years ago.

10. What is your current weight?
I weigh ___ kg.

11. What is your current height?
I am ___ cm tall.

12. How tall were you (as a young adult) around age 25?
I was about ___ cm tall.

13. What was your weight at around age 25?
I weighed about ___ kg.

14. How physically active are you? (Which statement best describes you?)
☐ I don’t walk at all. Completely wheelchair-bound.
☐ Mostly sedentary. Sometimes a short walk. Light housework such as reheating food, tidying, some vacuuming.
☐ Light exercise such as walking to the store several times a week, regular gardening. Do all light housework myself.
☐ More strenuous exercise such as brisk walks or gymnastics weekly. Do heavier gardening and housework.

15. Can you stand up from a regular chair 5 times in a row – without using your arms? Try it if you like.
☐ Yes, I can stand up 5 times in a row without using my arms
☐ No

16. Do you use any kind of walking aid? (You can check more than one box.)
☐ No
☐ Cane
☐ Walker
☐ Wheelchair
☐ Other: _________

17. Have you fallen in the past 12 months? How many times?
☐ No → skip to question 19
☐ Once
☐ Twice
☐ Three times
☐ Four times
☐ More than four times

18. Where did you fall? (You may check both boxes.)
☐ Indoors
☐ Outdoors

19. Have you broken any bones (fracture) after the age of 50?
☐ Yes
☐ No → skip to question 21

20. Where did you break a bone? (You may check several boxes.)
At what age (approximately)?
☐ Left hip at age ___
☐ Right hip at age ___
☐ Left wrist/forearm at age ___
☐ Right wrist/forearm at age ___
☐ Left upper arm at age ___
☐ Right upper arm at age ___
☐ Left ankle at age ___
☐ Right ankle at age ___
☐ Spine (compressed vertebra) at age ___
☐ Other fracture: ____________
☐ Don’t know

21. Do you have daily back pain?
☐ No
☐ Yes

22. Has your back ever been X-rayed? When?
☐ Yes, my back was X-rayed around (year): ___
☐ No → skip to question 23

Did the X-ray show any fracture?
☐ No, no vertebra was compressed
☐ Yes, one vertebra was compressed
☐ Yes, several vertebrae were compressed
☐ Don't know
☐ Other result: ____________

Would you like me to continue translating the rest of the questions (23–38 and medication list)?

Here is the continuation and **English translation of questions 23–38 and the medication section** from the HIP22 questionnaire:

---

23. Have you been told by a doctor that you have worn-out hip joints (osteoarthritis)?
☐ Yes
☐ No → skip to question 24

Have you been operated on for worn-out hip joints (osteoarthritis)? (You may check two boxes)
☐ Yes, left hip
☐ Yes, right hip
☐ No

24. Have you taken cortisone tablets (Prednisolone or Betapred) for more than three months in total?
☐ Yes
☐ No → skip to question 25
☐ Don't know → skip to question 25

For how long did you take cortisone tablets?
For approximately ___ months.

25. When did your menstruation stop?
I was ___ years old when my periods stopped.

26. During menopause or the years immediately after, did you take estrogen tablets such as Progynon, Kliogest, Trisekvens, or Cyklabil?

☐ Yes
☐ No → skip to question 30
☐ Don't know → skip to question 30

Which kind of estrogen? For how long did you use them?
Type of tablet: ____________
For approximately ___ years.

27. Have you given birth? If yes – how many children?

☐ No
☐ Yes, I have had ___ children

28. Have you breastfed any child for more than 9 months?

☐ Yes
☐ No

29. Have you ever had a stroke or bleeding in the brain?

☐ No
☐ Yes, but I no longer have any walking difficulties
☐ Yes, I was paralyzed and have walking difficulties afterwards
☐ Don't know

30. Do you see well?

☐ Yes, I see well (with or without glasses)
☐ No, I do not see well (even with glasses)

---

31. Have you been told by a doctor that you have any of the following conditions, or have you been operated on for any of them?

(You may check several boxes.)

☐ Osteoporosis (weakened bones or "osteoporosis")
☐ High blood pressure (medicated)
☐ Gluten intolerance (celiac disease)
☐ Rheumatoid arthritis (RA or "joint rheumatism")
☐ Parkinson's disease
☐ Asthma / chronic respiratory disease (medicated)
☐ Part of the stomach removed by surgery
☐ Uterus removed

☐ Both ovaries removed
☐ Other chronic illness: ______________________________
☐ No, I don’t have any of these conditions

---

32. Are any of your parents from a country outside the Nordic countries?
☐ Yes
☐ No

33. Living situation (You may check several boxes)
☐ Living alone
☐ Living with spouse/partner
☐ Living with another relative or friend
☐ Living in assisted housing or nursing home
☐ Other: ______________________________

34. Do you receive regular help at home?
☐ No
☐ Yes, from a relative or friend at least once per week
☐ Yes, from home care service daily
☐ Yes, from home care service 2–5 times per week
☐ Yes, from home care service once per week
☐ I live in assisted housing or nursing home and receive help as needed

35. Do you have regular contact with a doctor (at least once a year)?
☐ No
☐ Yes, I usually go to the health center / hospital
☐ Prefer not to answer

36. Do you take any medication daily?
☐ No
☐ Yes

37. Would you like us to contact you later if we believe you could benefit from special treatment or advice to prevent falls or fractures?
☐ Yes
☐ No

---

38. Medication list:

Please fill in the list below with the medications you usually take at least once a week (including over-the-counter medications, vitamins, laxatives, and herbal supplements). Write the name of the medication, the strength per tablet, and how many tablets you take per day or week.
If you have a prepared medication list, you can attach a copy instead of writing them out.

Examples:

- Alvedon, 500 mg per tablet, 2–6 tablets per day
- Seloken ZOC, 50 mg per tablet, 1 tablet per day
- Imovane, 5 mg per tablet, 1 tablet 1–2 times per week
- Laxoberal drops, 7.5 mg/mL, 12 drops 2 times per week

If you have any questions or suggestions, feel free to write a few lines here:

THANK YOU FOR YOUR PARTICIPATION –
WE HOPE IT BENEFITS YOUR FUTURE HEALTH!

Please return the form in the enclosed prepaid envelope to:
District Physician Daniel Albertsson
Vislanda Health Center, Gröna gatan 8–10, 340 30 Vislanda

Let me know if you’d like a clean, formatted version of the whole translated questionnaire as a Word or PDF document.
