# Supplementary material for: Reduced Risk of Recurrent Fragility Fractures After a Primary Care–Based Fracture Prevention Intervention: A 20-Year Non-Randomized Controlled Follow-Up Study in Women Aged 70–100
Source: Scand J Prim Health Care. 2025 Nov 6;44(1):1–16. doi: 10.1080/02813432.2025.2571929 (PMC12918357; doi:10.1080/02813432.2025.2571929)
Supplement: walk_well_original_2015.pdf [file IPRI_A_2571929_SM4450.pdf]

Om du vill veta mer – kontakta din vårdcentral, hemsjukvården eller ring 1177 om:

- promenad- och gymnastikgrupper inom området
- balans- och styrketräning med sjukgymnast
- halkskydd eller höftskydd
- hur du mer kan förebygga benbrott och fall

REGION KRONOBERG

1177 VÅRDGUIDEN

© Framtagen november 1998, Vislanda, reviderad av Daniel Albertsson, september 2015, Växjö

REGION KRONOBERG

GÅ BRA

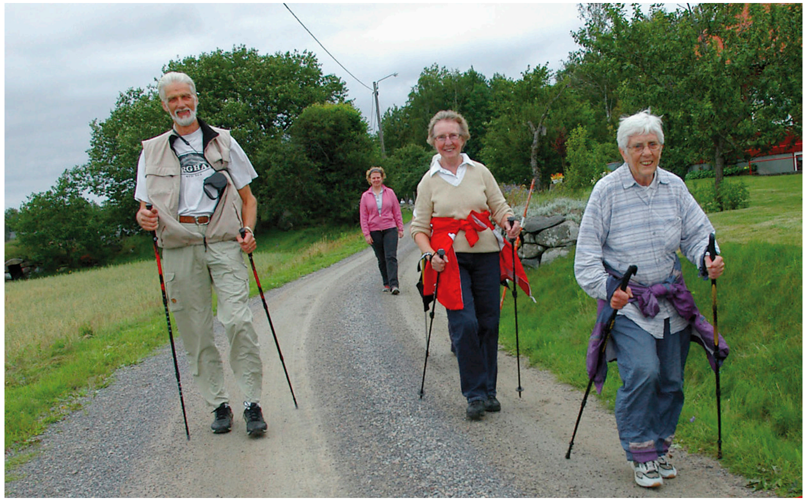

Foto: Matilda Andersson

Goda råd

- En daglig promenad gör nytta. Den stärker dina muskler och benstomme, motverkar yrsel och ger bra allmän kondition. När du är ute (under sommarhalvåret) bildas D-vitamin i huden. D-vitamin bevarar benstommen.

- Enkel träning hemma eller i grupp förbättrar din rörlighet och minskar fallrisken. Om du brukar ramla – börja rörelseträna och gör hemmiljön säkrare.

- Ät allsidigt och minst ett lagat mål mat om dagen, gärna rikligt med fibrer, grönsaker och frukt.
  Ost och mjölk är rika på kalk och dagsbehovet motsvaras av ett glas mjölk, en tallrik yoghurt samt fyra skivor ost. Mager mjölk, med fetthalt 1,5 % eller lägre, är vitamin D-berikad och är lika kalkrik som fet mjölk.

- Gott humör, kropp och själ hör ihop. Har du ont eller är orolig för något kan du må sämre. Isolera dig inte utan sök trevligt umgänge och något att intressera dig för så behåller du hälsan bättre.

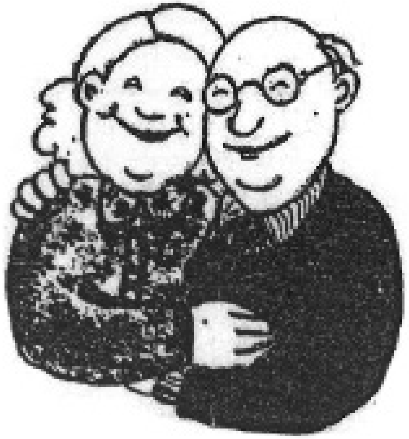

	JA	NEJ
• Jag promenerar dagligen	☐	☐
• Jag tränar balans och muskelstyrka hemma varje vecka	☐	☐
• Jag tränar balans och muskelstyrka i grupp varje vecka	☐	☐
• Jag använder reflexer ute vid skymning och mörker	☐	☐
• Jag använder halkfria skor eller halkskydd när jag vistas ute vintertid	☐	☐
• Jag går bättre med gånghjälpmedel, har rollator, stavar eller käpp (har isdubb)	☐	☐
• I trappor finns god belysning, halkskydd på trappsteg samt stadiga ledstänger	☐	☐

	JA	NEJ
• Jag har halkmatta i badkar, dusch och på badrumsgolvet	☐	☐
• Jag har handtag på väggen som stöd vid badkar och toalett	☐	☐
• Jag sitter ner när jag duschar	☐	☐
• Föremål som jag använder ofta har jag lättillgängligt (ej högt upp)	☐	☐
• Jag undviker att klättra på stolar och pallar mm	☐	☐
• Jag har god belysning i bostad och nattlyse om jag går upp nattetid	☐	☐
• Jag en telefon vid sängen eller en bärbar telefon	☐	☐
• Jag använder höftskydd eftersom jag ramlar ofta och har brutit mig tidigare	☐	☐
• Jag äter en allsidig och näringsrik kost, där ost och mjölk ingår	☐	☐

Snubbla och halka är vanligt − visste du att...

- fall är den vanligaste orsaken till svårare kroppsskada.
- höftfraktur drabbar ofta äldre personer och oftare kvinnor än män.
- antalet fallolyckor har ökat. Detta beror på att vi blir äldre, men också på att vi rör oss mindre.
- de flesta fallskadorna inträffar i hemmet. Det beror ofta på att man snubblar eller halkar.
- fall kan förebyggas genom att rörelseträna hemma och att promenera mer samt genom att minska fallriskerna i hemmet.
- kroppsundersökning kan behövas vid upprepade fall. Trötthet, yrsel eller dålig syn kan öka fallrisken.
- om du faller ofta och har brutit dig tidigare kan du förebygga höftfraktur genom att använda höftskydd.
- god kondition gör att man återhämtar sig fortare, även efter en svårare kroppsskada.

Många skador kan förebyggas

- Ta bort lösa mattor, där du går ofta.

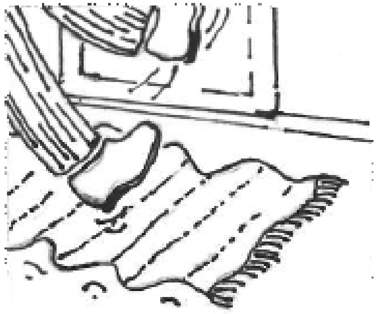

- Använd halkskydd under de mattor du vill ha kvar. Skyddet behöver bytas efter några år.

- En bit heltäckningsmatta med gummerad undersida är ännu mer halkfri.

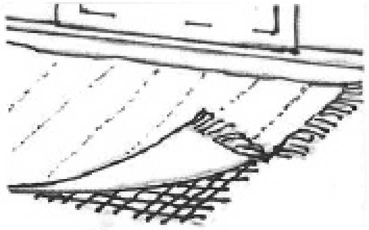

- Fäst upp elsladdar så att de inte ligger lösa på golvet.

- Ha bra belysning i bostaden (är lampstyrkan tillräcklig?)

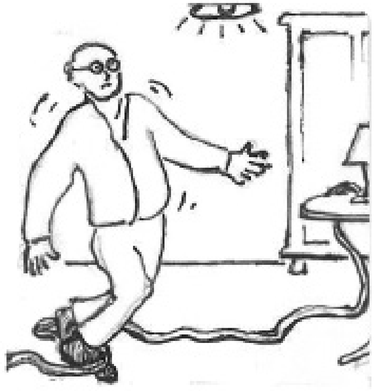

Checklista

	JA	NEJ
• Jag har tagit bort onödiga mattor	☐	☐
• Jag har halkskydd under lösa mattor	☐	☐
• Jag använder stadiga skor inne med hälkappa och bra sulor, eller plasti-socks	☐	☐
• Jag har fäst upp sladdar så att de inte ligger lösa på golvet	☐	☐
• Jag undviker att polera golv för att minska halkrisk	☐	☐
• Jag har tagit bort trösklar i bostaden	☐	☐
• Jag har god belysning i bostaden	☐	☐
• Jag har kollat synen och använder bra glasögon	☐	☐
• Jag sitter vid av- och påklädning	☐	☐

Om du ramlar

Det kan vara bra att veta hur du tar dig upp själv om du ramlar. Öva gärna när någon person är närvarande som kan hjälpa dig. Handla med eftertanke om du ramlar. Försök komma upp från golvet, se bilder nedan. Annars, försök nå en filt som håller dig varm. Försök göra oväsen, som att knacka i golvet med en käpp. En bärbar telefon som du alltid har med dig underlättar att ringa efter hjälp.

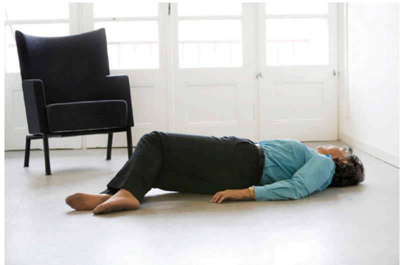

1. Rulla över på sidan och res dig upp till sittande på golvet.

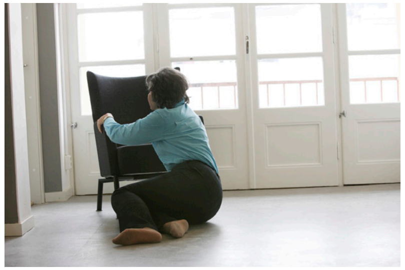

2. Ta dig fram till en stadig möbel, till exempel en fåtölj eller en soffa, genom att hasa på rumpan eller krypa. Ta tag i möbeln och försök att komma upp på knä.

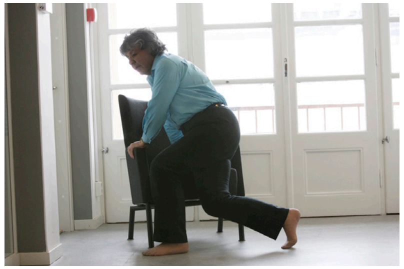

3. Tryck dig upp med ditt starkaste ben och din starkaste arm medan du fortfarande håller dig i möbeln.

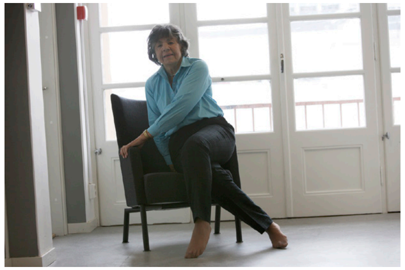

4. Sätt dig ner på möbeln.

Foto: Tommy Andersson

- Synen bör undersökas regelbundet, gärna årligen. Använd rätt glasögon!

- Ta det alltid lugnt då du stiger upp från sängen. Om du brukar bli yr, sitt kvar en stund och trampa med fötterna mot golvet. ”Morgongymnastik” i sängen kan vara bra.

- Ordna så att du har en telefon lättillgänglig från sängen, eller en bärbar telefon.

- Behöver du gå upp på toaletten under natten så använd nattbelysning. Kontakta gärna hemsjukvården om praktiska hjälpmedel som kan underlätta.

- Sitt ned vid av- och påklädning om du är ostadig.

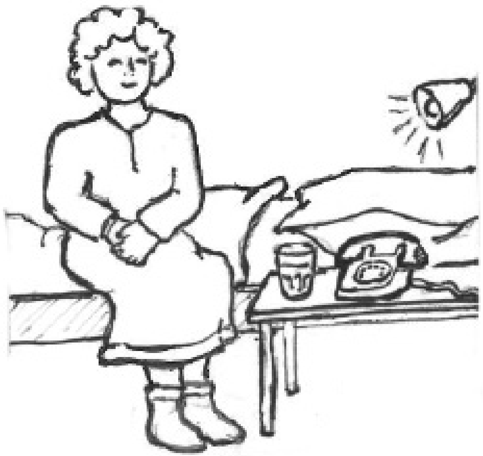

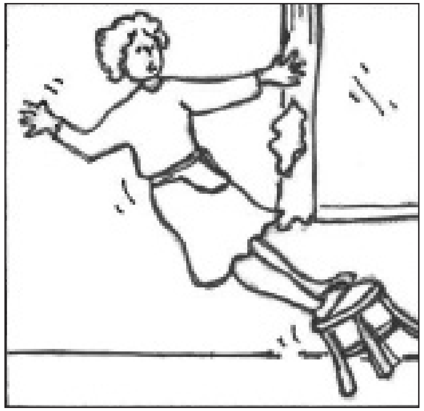

- Saker som du använder ofta, exempelvis köksredskap, bör du förvara lättåtkomligt, inte högt upp i skåpen.

- Be om hjälp med gardinbyten, lampbyten och annat svåråtkomligt hemarbete.

- Skaffa en stadig stege med handstöd.

- Stå aldrig på ostadiga stolar eller pallar.

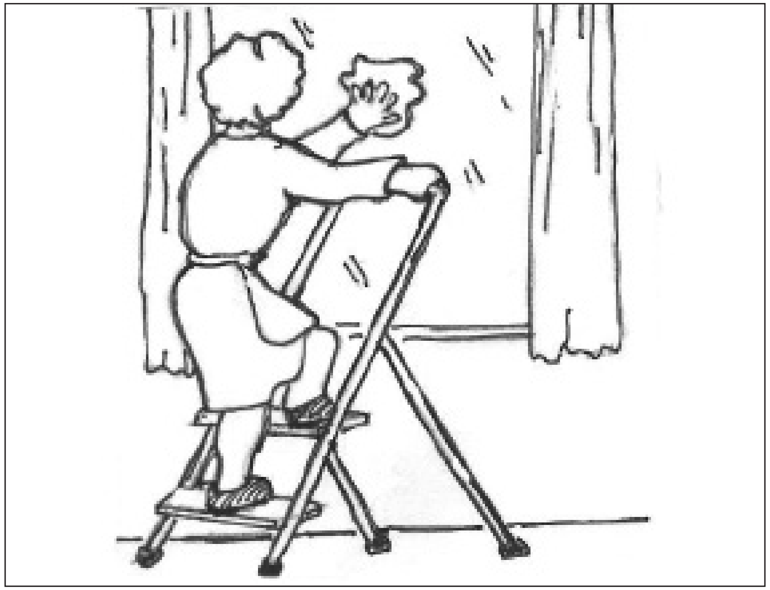

Stå i ett hörn vänd ut mot rummet med en stolsrygg framför dig

- Träna att stå med fötterna tätt ihop under någon minut, försök blunda.
- Gör som ovan, men stå på en skumgummidyna.
- Försök stå på ena benet på golvet, seende.

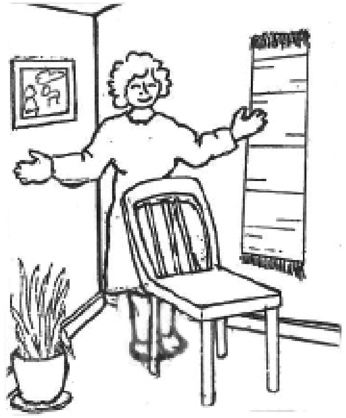

Håll dig i stolsryggen och

- gå upp på tå 5–10–15–20 gånger. Öka efterhand.
- gör djupa knäböjningar. Öka efterhand.
- försök göra ovanstående övningar utan att hålla i stolen.

Gör alla rörelser långsamt och lugnt. Tänk på hur du upplever dina rörelser i kroppen medan du gör dem!
Börja träna dagligen i 1-2 månader. Fortsätt träna, minst 3 gånger i veckan.

Träningsråd för att få bättre balans och muskelstyrka

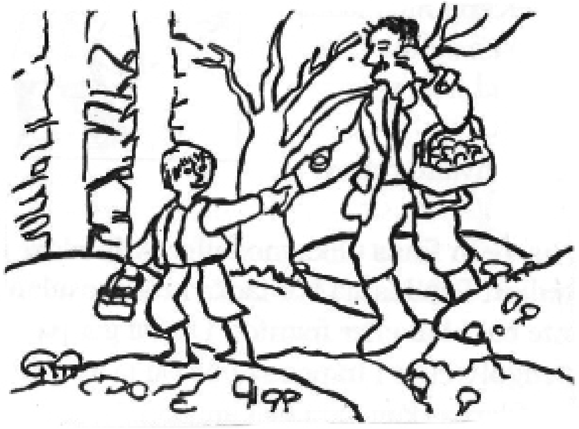

- En promenad på 30 minuter varje dag ger bra motion (kan även delas upp i flera kortare pass). Gå gärna i rask takt och på ojämnt underlag. Stavgång tränar både ben och armar och ryggen rätas ut. Gymnastik, dans och trädgårdsarbete är också bra.

- Om du är ovan vid att gå, kan du börja med korta promenader. Även enklare övningar gör nytta för otränade muskler. Ork och gång brukar förbättras redan efter 1-2 månader.

- Om du har svårt för att promenera ute, kan du skaffa en träningscykel för inomhusbruk. Öka belastningen långsamt och efter förmåga. Träna 15-20 minuter varje dag.

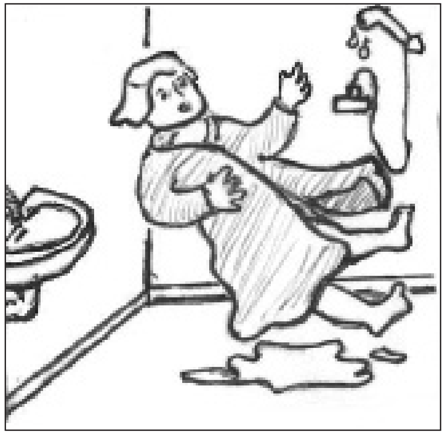

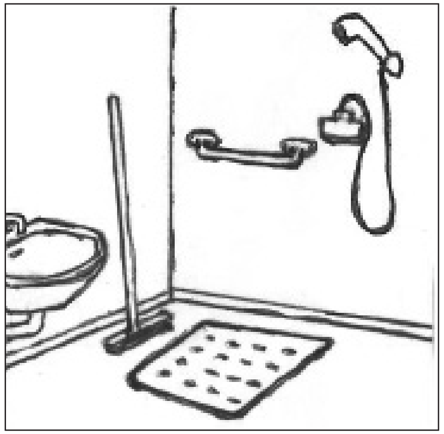

- Lägg gummihalkskydd i badkar och dusch. Sitt gärna på en duschpall när du duschar.

- Lägg en halkfri matta på golvet som du kan sätta fötterna på när du badat eller duschat.

- Montera handtag som du kan hålla i vid badkar, dusch och toalett.

- Kom ihåg – våta golv är hala golv. Använd golvskrapa.

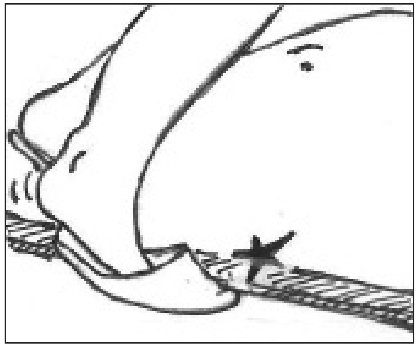

- Använd inneskor som sitter ordentligt på fötterna, med hälkappa och bra sulor.
  Alternativ är plastisocks (sockor med gummibelagd undersida).

- Gå aldrig i strumplästen.

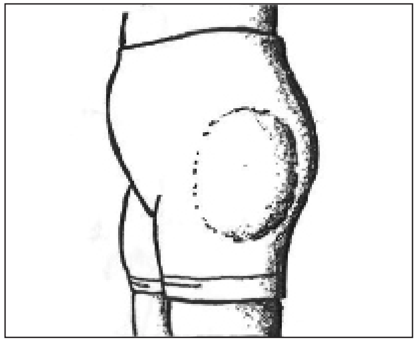

- Ta bort trösklar som inte är nödvändiga. Hör med värd och hemsjukvård före.

- Höftskydd kan förebygga brott i höften. Om du ramlar ofta – hör med hemsjukvården eller din vårdcentral.

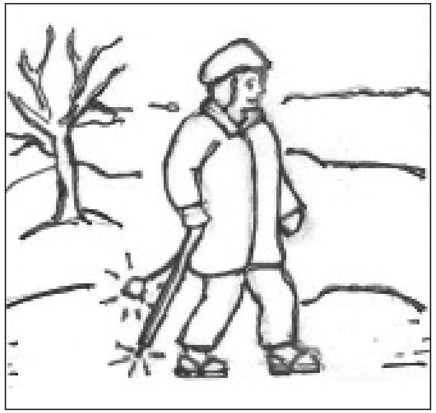

- Använd stadiga skor ute. Broddar behövs vid halt väglag.

- Använd reflex.

- Gånghjälpmedel som rollator, stavar och käpp kan förbättra gång.

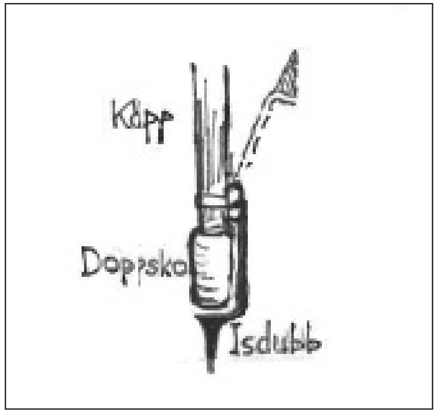

- Om du använder käpp så behövs isdubb och doppsko. Det kan du erhålla hos din vårdcentral eller hemsjukvården.
  Kom ihåg att fälla upp isdubb inomhus! Byt en sliten doppsko!

- Lösa halkskydd finns på apotek, skoaffär eller varuhus.
  Skydd ska sitta stadigt. De bör vara lätta att ta av och på.
  Bra om broddarna täcker hela fotsulan.
  Ta gärna av dig broddarna då du går på stengolv eller sten-trappa eftersom de blir hala mot hårt underlag.
