# Supplementary material for: Reduced Risk of Recurrent Fragility Fractures After a Primary Care–Based Fracture Prevention Intervention: A 20-Year Non-Randomized Controlled Follow-Up Study in Women Aged 70–100
Source: Scand J Prim Health Care. 2025 Nov 6;44(1):1–16. doi: 10.1080/02813432.2025.2571929 (PMC12918357; doi:10.1080/02813432.2025.2571929)
Supplement: Figure 1A.pptx [file IPRI_A_2571929_SM4449.pptx]

## Slide 1
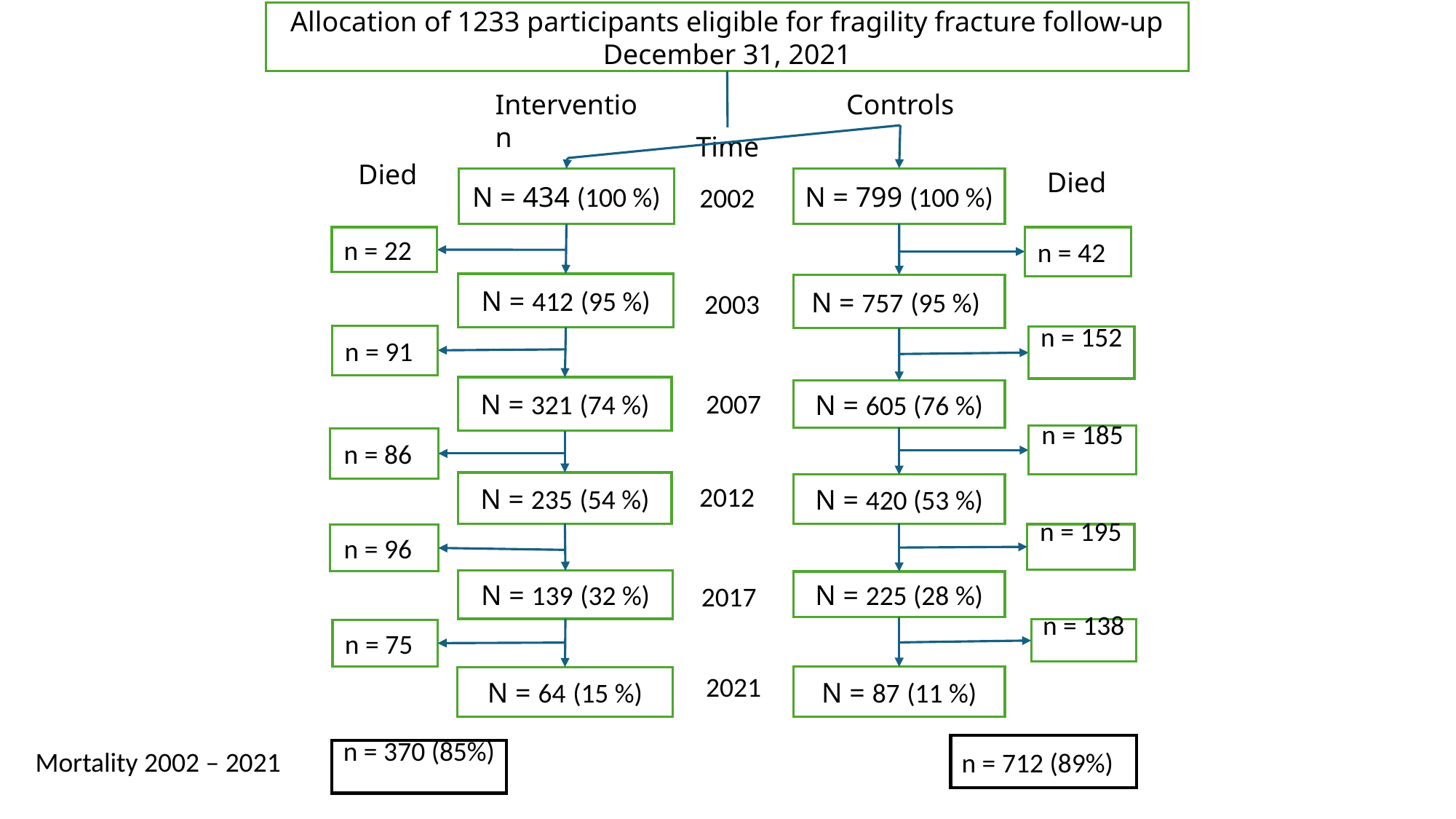

Allocation of 1233 participants eligible for fragility fracture follow-up December 31, 2021
Intervention
Controls
Time
Died
Died
N = 799 (100 %)
N = 434 (100 %)
2002
n = 22
n = 42
 N = 412 (95 %)
N = 757 (95 %)
2003
n = 91
n = 152
 N = 321 (74 %)
2007
 N = 605 (76 %)
n = 185
n = 86
2012
 N = 235 (54 %)
 N = 420 (53 %)
n = 195
n = 96
 N = 139 (32 %)
2017
 N = 225 (28 %)
n = 138
n = 75
2021
 N = 87 (11 %)
 N = 64 (15 %)
Mortality 2002 – 2021
n = 712 (89%)
n = 370 (85%)
