# Supplementary material for: Reduced Risk of Recurrent Fragility Fractures After a Primary Care–Based Fracture Prevention Intervention: A 20-Year Non-Randomized Controlled Follow-Up Study in Women Aged 70–100
Source: Scand J Prim Health Care. 2025 Nov 6;44(1):1–16. doi: 10.1080/02813432.2025.2571929 (PMC12918357; doi:10.1080/02813432.2025.2571929)
Supplement: Survey_HIP22_baseline_Swedish.pdf [file IPRI_A_2571929_SM4448.pdf]

Vårdcentralen
Vislanda
Landstinget Kronoberg

01-09-23

Har Du risk för höftfraktur?[1]

Tack för att Du deltog i den förra undersökningen om benskörhet och fall. Vi hoppas att Du har användning av de råd som givits. Undersökningen kommer att utvärderas under ytterligare några år varefter vi meddelar mer resultat.

Då intresset varit stort så har vi beslutat att skicka ut en ny enkät till alla kvinnor över 70 år i Vislandaområdet. Det är värdefullt om Du kan delta även i denna undersökning.

Benskörhet (osteoporos) är en sjukdom med nedsatt bentäthet och ökad risk för benbrott. Sjukdomen blir allt vanligare, en kvinna idag har dubbelt så stor risk för benbrott jämfört med på 1950-talet. De vanligaste benbrotten är brott på underarm, ryggkotor och lårbenshals (höftfraktur).

För att minska risken för benbrott räcker det för många med goda vardagsvanor. Om man till exempel promenerar dagligen stärks muskler och skelett och balansen förbättras. Man kan också minska risken för fall genom att göra hemmiljön säkrare eller använda halkskydd. Om man har hög risk för att falla finns det höftskydd att bära. En del kan behöva kalciumtillskott i kosten eller andra läkemedel för att stärka skelettet.

Vi vill i den här undersökningen kartlägga risken för benskörhet och fall hos äldre kvinnor i hela Vislandaområdet.

---

[1] Meof8029-3-6-enk-följ-00-Vis92-led/010907

Du är en av de kvinnor över 70 år som blivit utvald att delta.

Om Du väljer att delta ber vi Dig fylla i bifogat frågeformulär och sända det till oss i svarskuvertet. Om Du skulle behöva hjälp att fylla i formuläret går det bra att be någon anhörig eller personal från hemtjänsten om hjälp.

Efter att vi mottagit Ditt formulär kommer vi att sända Dig en kortfattad information med råd. Vi kommer att kontakta alla som vi tror kan ha nytta av någon särskild behandling eller ytterligare rådgivning. Vi ber Dig ange i frågeformuläret på sidan 5 (fråga 37) om Du i så fall vill bli kontaktad av sjuksköterska senare.

Informationen från frågeformuläret databearbetas och handhas enligt sjukvårdens sekretesslagar.

Din medverkan är helt frivillig och Du kan när som helst avbryta Ditt deltagande utan att det påverkar annan behandling.
Genom att Du deltar i undersökningen kan Du minska risken att falla och få ett benbrott.

Dina svar är värdefulla för undersökningen även om Du mår helt bra.

Om Du har några frågor så går det bra att ringa till sjuksköterska (se nedan) eller till Daniel Albertsson på Vislanda vårdcentral (0472/30154).

Vänliga hälsningar

Daniel Albertsson
Projektansvarig. Läkare

Roland Johansson
Vårdc.chef. Läkare

Mats Henriksson
Läkare

Eva Hjerpe
Läkare

Agneta Lönn
Sjuksköt. Vislanda Vårdc.

Annika Magnusson
Sjuksköt. Björkliden

Maria Lindahl
Sjuksköt. Asken & Torsgården

	Besöksadress	Postadress	Telefon	e-mail
Vårdcentralen Vislanda	Gröna gatan 8-10	340 30 Vislanda	0472-301 54	daniel.albertsson@ltkronoberg.se
Björkliden	Sockenv. 15	340 30 Vislanda	0472-35055	
Asken	Hantverksg. 4	340 32 Grimslöv	0470-750069	
Torsgården	Lönashult	340 30 Vislanda	0470-757011	

Har Du risk för höftfraktur?[1]

Information om undersökning om risk för benskörhet och fall hos kvinnor över 70 år i Vislanda området

Benskörhet (osteoporos) är en sjukdom med nedsatt bentäthet och ökad risk för benbrott. Sjukdomen blir allt vanligare, en kvinna idag har dubbelt så stor risk för benbrott jämfört med på 1950-talet. De vanligaste benbrotten är brott på underarm, ryggkotor och lårbenshals (höftfraktur).

För att minska risken för benbrott räcker det för många med goda vardagsvanor. Om man till exempel promenerar dagligen stärks muskler och skelett och balansen förbättras. Man kan också minska risken för fall genom att göra hemmiljön säkrare eller använda halkskydd. Om man har hög risk för att falla finns det höftskydd att bära. En del kan behöva kalciumtillskott i kosten eller andra läkemedel för att stärka skelettet.

Vi vill i den här undersökningen kartlägga risken för benskörhet och fall hos äldre kvinnor i Vislanda området.

Du är en av de kvinnor över 70 år som blivit utvald att delta.

Om Du väljer att delta ber vi Dig fylla i bifogat frågeformulär och sända det till oss i svarskuvertet.

---

[1] Meof8029-3-6-enk-följ-00-Vis350-led/010923

Om Du skulle behöva hjälp att fylla i formuläret går det bra att be någon anhörig eller personal från hemtjänsten om hjälp.

Efter att vi mottagit Ditt formulär kommer vi att sända Dig en kortfattad information med råd. Vi kommer att kontakta alla som vi tror kan ha nytta av någon särskild behandling eller ytterligare rådgivning. Vi ber Dig ange i frågeformuläret på sidan 5 (fråga 37) om Du i så fall vill bli kontaktad av sjuksköterska senare.

Informationen från frågeformuläret databearbetas och handhas enligt sjukvårdens sekretesslagar.

Din medverkan är helt frivillig och Du kan när som helst avbryta Ditt deltagande utan att det påverkar annan behandling.

Genom att Du deltar i undersökningen kan Du minska risken att falla och få ett benbrott.

Dina svar är värdefulla för undersökningen även om Du mår helt bra.

Om Du har några frågor så går det bra att ringa till sjuksköterska (se nedan) eller till Daniel Albertsson på Vislanda vårdcentral (0472/30154).

Vänliga hälsningar

Daniel Albertsson
Projektansvarig. Läkare

Roland Johansson
Vårdc.chef. Läkare

Mats Henriksson
Läkare

Eva Hjerpe
Läkare

Agneta Lönn
Sjuksköt. Vislanda Vårdc.

Annika Magnusson
Sjuksköt. Björkliden

Maria Lindahl
Sjuksköt. Asken & Torsgården

	Besöksadress	Postadress	Telefon	e-mail
Vårdcentralen Vislanda	Gröna gatan 8-10	340 30 Vislanda	0472-301 54	daniel.albertsson@ltkronoberg.se
Björkliden	Sockenv. 15	340 30 Vislanda	0472-35055	
Asken	Hantverksg. 4	340 32 Grimslöv	0470-750069	
Torsgården	Lönashult	340 30 Vislanda	0470-757011	

Vårdcentralen Vislanda
Landstinget Kronoberg

....

Namn
Adress

Personnummer : ....

Telefon (fyll gärna i) :..............................

Frågor till kvinnor över 70 år om benskörhet och fall [1]

Markera med kryss i en av rutorna på var fråga - om det inte anges något annat.
Kryssa för den ruta som passar Dig bäst även om den inte stämmer helt.
Dom små markerings-siffrorna (1,2,3) i formuläret behöver Du inte bry Dig om.
Om du undrar eller vill lägga till något så skriv lite bredvid frågan eller ring oss.

1. **Hur många år är Du nu ?**

   Jag är .... år .

2. **Hur mår Du nu för tiden ?**

   - ❑ 1. Utmärkt.
   - ❑ 2. Rätt så bra
   - ❑ 3. Dåligt.

3. **Har Din mor drabbats av benbrott av höften (fraktur av lårbenshals) ?**

   - ❑ 1. Ja. (Hon var ungefär 4............år då.)
   - ❑ 2. Nej.
   - ❑ 3. Vet inte.

4. **Har någon av dina föräldrar eller syskon drabbats av benbrott på underarm, överarm, kota eller höft under vuxen ålder?**

   - ❑ 1. Ja, ...
   - ❑ 2. Nej.
   - ❑ 3. Vet inte.

5. **Hur mycket mjölk, fil eller youghort får Du i Dig om dan ?**

   Jag dricker .... stycken glas mjölk eller tallrikar fil om dan.

6. **Hur många koppar kaffe brukar Du dricka varje dag ?**

   Jag dricker ungefär .... koppar kaffe om dan.

7. **Hur många skivor ost brukar Du äta varje dag ?**

   Jag äter ungefär .... stycken skivor ost om dan.

---

meof8028-4-6-4Xenk-00-4-led-3-5/ 01-09-23

8. Röker Du numera?

- ❑ 1. Ja.
- ❑ 2. Nej, men jag har rökt förut.
- ❑ 3. Nej, jag har aldrig rökt → gå vidare till fråga 10.

9. Hur mycket har Du rökt?

Jag började röka vid 1......... års ålder. Jag slutade röka för 2........ år sen.

10. Vad är Din nuvarande vikt?

Jag väger .... kg.

11. Hur lång är Du nu ?

Jag är .... cm lång.

12. Hur lång var Du (som längst) - då Du var ungefär 25 år ?

Jag var ungefär .... cm lång.

13. Vad vägde Du som ung - då Du var kring 25 årsåldern ?

Jag vägde ungefär ...... kg då.

14. Hur mycket brukar du röra Dig ? (- Vilket påstående passar bäst in på Dig ?)

- ❑ 1. Går inte något. Helt rullstolsbunden.
- ❑ 2. Mestadels stillasittande. Ibland någon kort promenad. Lätt hushållsarbete såsom uppvärmning av mat, undanplockning och lite dammsugning.
- ❑ 3. Lättare ansträngning som promenader till affär flera gånger per vecka, vanligt trädgårdsarbete. Gör allt lättare hushållsarbete själv typ matlagning, dammsugning mm.
- ❑ 4. Mer ansträngande motion med längre raska promenader eller gymnastik var vecka. Gör tyngre trädgårdsarbete och hushållsarbete.

15. Kan Du resa Dig upp från en vanlig stol 5 gånger i följd - <u>utan</u> att hjälpa till med armarna? Pröva gärna.

- ❑ 1. Ja, jag kan resa mig upp 5 gånger i följd utan att hjälpa till med armarna.
- ❑ 2. Nej.

16. Använder Du någon typ av gånghjälpmedel ? (Här kan du kryssa för flera rutor.)

- ❑ 1. Nej.
- ❑ 2. Käpp.
- ❑ 3. Rollator.
- ❑ 4. Rullstol.
- ❑ 5. Annat ....

17. Har Du ramlat omkull de sista 12 månaderna ?
Hur många gånger ?

- ☐ 1. Nej → gå vidare till fråga 19.
- ☐ 2. En gång.
- ☐ 3. Två gånger.
- ☐ 4. Tre gånger.
- ☐ 5. Fyra gånger.
- ☐ 6. Mer än fyra gånger.

18. Var ramlade Du då ? (Här får man kryssa för båda rutorna.)

- ☐ 1. Inomhus.
- ☐ 2. Utomhus.

19. Har Du brutit något ben i kroppen (fraktur) **efter att Du fyllt 50 år ?**

- ☐ 1. Ja.
- ☐ 2. Nej → gå vidare till fråga 21.

20. Var har Du då brutit Dig ? (Här kan Du kryssa i flera rutor.)
Vid **vilken ålder** bröt Du dig (ungefär) ?

☐	1. Lårbenshals vänster sida vid ålder ....
☐	2. Lårbenshals höger sida vid ålder ....
☐	3. Handled / underarm vä. vid ålder ....
☐	4. Handled / underarm hö vid ålder ....
☐	5. Överarm vä vid ålder ....
☐	6. Överarm hö vid ålder ....
☐	7. Fotled vä vid ålder ....
☐	8. Fotled hö vid ålder ....
☐	9. Ryggkota ihoptryckt vid ålder ....
☐	10. Annat benbrott ....
☐	11. Vet ej.

21. Har Du daglig ryggvärk?

- ☐ 1. Nej.
- ☐ 2. Ja.

22. Har ryggen blivit röntgad? När då?

- ☐ 1. Ja, ryggen blev röntgad .... (ange ungefär när).
- ☐ 2. Nej → gå vidare till fråga 23.

Visade ryggröntgen någon fraktur då?

- ☐ 3. Nej, ingen ryggkota var hoptryckt.
- ☐ 4. Ja, en ryggkota var hoptryckt.
- ☐ 5. Ja, flera kotor var hoptryckta.
- ☐ 6. Vet ej svaret på röntgen.
- ☐ 7. Annat besked ....

23. Har Du av läkare fått veta att du har utslitna höfter (artros) ?

- ❑ 1. Ja.
- ❑ 2. Nej → gå vidare till fråga 24.

Är Du då opererad för <u>utslitna</u> höftleder (artros)? (Här kan Du fylla i två rutor)

- ❑ 3. Ja, på vänster höft.
- ❑ 4. Ja, på höger höft.
- ❑ 5. Nej.

24. Har du tagit kortisontabletter (Prednisolon eller Betapred) under sammanlagt mer än tre månader ?

- ❑ 1. Ja.
- ❑ 2. Nej → gå vidare till fråga 25.
- ❑ 3. Vet ej → gå vidare till fråga 25.

Under hur lång tid har Du då tagit kortisontabletter ?
Under ungefär 4............ månader.

25. När upphörde Dina menstruationer ?
Jag var .... år när menstruationerna upphörde.

26. Har Du under klimakteriet eller åren direkt därefter tagit östrogentabletter av sorten Progynon, Kliogest, Trisekvens, Cyklabil.

- ❑ 1. Ja
- ❑ 2. Nej → gå vidare till fråga 30.
- ❑ 3. Vet ej → gå vidare till fråga 30.

Vilken sorts östrogen ? **Hur länge använde Du dem ?**
Tablettsort: 4........................ under 5........... år.

27. Har Du fött barn? I så fall – hur många barn?

- ❑ 1. Nej.
- ❑ 2. Ja, jag har fått 3 .... barn.

28. Har Du ammat något barn mer än 9 månader?

- ❑ 1. Ja.
- ❑ 2. Nej.

29. Har Du fått någon propp eller blödning i hjärnan ?

- ❑ 1. Nej.
- ❑ 2. Ja, men jag har inga gångsvårigheter längre.
- ❑ 3. Ja. Jag är förlamad och har gångsvårigheter efteråt.
- ❑ 4. Vet ej.

30. Ser Du bra ?

- ❑ 1. Ja. jag ser bra (med eller utan glasögon.)
- ❑ 2. Nej, jag ser inte bra (även om jag har glasögon.)

31. Har Du av läkare fått veta att Du har någon av följande sjukdomar eller har Du blivit opererad härför ? (Här kan du kryssa för flera rutor)

❑	1. Benskörhet (urkalkat skelett eller "osteoporos").
❑	2. Högt blodtryck (läkemedelbehandlas).
❑	3. Glutenintolerans (celiaci).
❑	4. Reumatoid artrit (RA eller "ledgångsreumatism").
❑	5. Parkinson.
❑	6. Astma / kronisk luftvägssjukdom (läkemedelbehandlas).
❑	7. Delar av magsäcken är bortopererad.
❑	8. Livmodern är bortopererad.
❑	9. Båda äggstockarna är bortopererade.
❑	10. Annan långvarig sjukdom: ....
❑	11. **Nej.** Jag har inte någon av dessa sjukdomar.

32. Kommer någon av Dina föräldrar från ett annat land än de nordiska länderna ?

❑ 1. Ja.
❑ 2. Nej.

33. Boende (Här kan du kryssa flera rutor)

❑ 1. Ensamboende.
❑ 2. Sammanboende / bor med make.
❑ 3. Bor med annan släkting eller vän.
❑ 4. Bor på servicehus eller sjukhem.
❑ 5. Annat, nämligen ....

34. Får Du regelbunden hjälp i hemmet ?

❑ 1. Nej.
❑ 2. Ja, av anhörig eller bekant minst 1 gång per vecka.
❑ 3. Ja, av hemtjänst var dag.
❑ 4. Ja, av hemtjänst 2 - 5 gånger per vecka.
❑ 5. Ja, av hemtjänst 1 gång per vecka.
❑ 6. Bor på servicehus eller sjukhem och får hjälp vid behov.

35. Har Du regelbunden kontakt med någon läkare (minst en gång per år) ?

❑ 1. Nej.
❑ 2. Ja, jag brukar gå till ....vårdcentral / lasarett.
❑ 3. Avstår från att svara på frågan.

36. Tar Du någon medicin dagligen?

❑ 1. Nej.
❑ 2. Ja.

37. Vill Du att vi kontaktar Dig senare om vi tror att Du har nytta av någon särskild behandling eller råd för att förebygga fall eller benbrott?

❑ 1. Ja.
❑ 2. Nej.

Fyll nu gärna i **listan nedan med de läkemedel Du brukar ta minst 1 gång per vecka** (även receptfria mediciner, vitaminer, laxermedel och hälsokost preparat).
Skriv tablettsort , styrkan på 1 tablett, och hur många tabletter du sammanlagt brukar ta under ett helt dygn eller under 1 vecka. Om du har en färdig **läkemedelslista** så kan du bifoga en kopia istället för att skriva ner dina mediciner.

Till exempel kan du skriva :
Alvedon, 500 mg per tablett, 2-6 tabletter per dygn
Seloken zoc, 50 mg per tablett, 1 tablett per dygn
Imovane, 5 mg per tablett, 1 tablett 1-2 ggr i veckan
Laxoberal droppar, 7,5 mg per ml, 12 droppar 2 ggr per vecka.

38. Läkemedelslista :

____
____
____
____
____
____
(Om listan inte räcker till så fortsätt på ett vanligt blad.)

Om Du har frågor eller förslag så skriv gärna några rader här :

____
____
____

**TACK FÖR DIN MEDVERKAN –
VI HOPPAS ATT DET GAGNAR DIN FRAMTIDA HÄLSA !**

Skicka formuläret i bifogat kuvert (portot är betalt) till Distriktsläkare Daniel Albertsson, Vårdcentralen Vislanda, Gröna gatan 8-10, 340 30 Vislanda.

Landstinget Kronoberg

Landstinget i Kalmar Län

Hur mår ditt skelett? [2]

Information om undersökning om risk för benskörhet och fall hos kvinnor över 70 år i Emmabodaområdet

Benskörhet (osteoporos) är en sjukdom med nedsatt bentäthet och ökad risk för benbrott. Sjukdomen blir allt vanligare, en kvinna idag har dubbelt så stor risk för benbrott jämfört med på 1950-talet. De vanligaste benbrotten är brott på underarm, ryggkotor och lårbenshals(höftfraktur).

För att minska risken för benbrott räcker det för många med goda vardagsvanor. Om man till exempel promenerar dagligen stärks muskler och skelett och balansen förbättras. Man kan också minska risken för fall genom att göra hemmiljön säkrare eller använda halkskydd. Om man har hög risk för att falla finns det höftskydd att bära. En del kan behöva kalciumtillskott i kosten eller andra läkemedel för att stärka skelettet.

Vi vill i den här undersökningen kartlägga risken för benskörhet och fall hos äldre kvinnor i Emmabodaområdet. Genom dina svar får vi mer kunskap för att på sikt bättre kunna förebygga benbrott.

Du är en av de kvinnor över 70 år som blivit utvald att delta.

Om Du väljer att delta ber vi Dig fylla i bifogat frågeformulär och sända det till oss i svarskuvertet.

Om Du skulle behöva hjälp att fylla i formuläret går det bra att be någon närstående eller vårdpersonal från hemtjänsten om hjälp.

[2] Meof8029-3-6-enk-följ-00-Emma-2/010902

Informationen från frågeformuläret databearbetas och handhas enligt gällande sekretessbestämmelser. Resultatet av undersökningen kommer att presenteras avidentifierat – det går då inte att känna igen svaren från någon särskild person.

Din medverkan är helt frivillig och Du kan när som helst avbryta Ditt deltagande utan att det påverkar Din nuvarande behandling.

Genom att Du deltar i undersökningen kan Du öka förståelsen om orsakerna för benbrott av höften och på sikt minska risken för att falla och bryta sig.

Dina svar är värdefulla för undersökningen även om Du mår helt bra.

Om Du har några frågor som rör formuläret eller undersökningen så går det bra att ringa till Daniel Albertsson på Vårdcentralen Vislanda (0472/30154) eller kontakta sjuksköterska på Din vårdcentral eller servicehus.

Vänliga hälsningar

Daniel Albertsson
Projektansvarig, läkare
Vårdcentralen Vislanda

Janti Gosai
Läkare
Vårdcentralen Emmaboda

Sune Blanking
Läkare
Läkarservice AB. Emmaboda

Eila Medin
Medicinskt ansvarig sjuksköt./enhetschef
Emmaboda kommun

Siv Öijerfeldt
Verksamhetschef
Distriktsvården Nybro/Emmaboda

	Besöksadress	Postadress	Telefon	e-mail
Vårdcentralen Vislanda	Gröna gatan 8-10	340 30 Vislanda	0472-301 54	daniel.albertsson@ltkronoberg.se
Vårdcentralen Emmaboda	Rådhusgatan 14	361 30 Emmaboda	0471-18500	
Läkarservice AB	Rådhusgatan 14	361 30 Emmaboda	0471-12400	
Emmaboda kommun	Järnvägsg. 12	361 21 Emmaboda	0471-18321	

EPN Bilaga 6

Landstinget Kronoberg

Landstinget i Kalmar Län

Namn
Adress

Personnummer : ....

Telefon (fyll gärna i) :..............................

Frågor till kvinnor över 70 år om benskörhet och fall [1]

Markera med kryss i en av rutorna på var fråga - om det inte anges något annat.
Kryssa för den ruta som passar Dig bäst även om den inte stämmer helt.
Dom små markerings-siffrorna (1,2,3) i formuläret behöver Du inte bry Dig om.
Om du undrar eller vill lägga till något så skriv lite bredvid frågan eller ring oss.

1. **Hur många år är Du nu ?**

   Jag är .... år .

2. **Hur mår Du nu för tiden ?**

   - ☐ 1. Utmärkt.
   - ☐ 2. Rätt så bra.
   - ☐ 3. Dåligt.

3. **Har Din mor drabbats av benbrott av höften (fraktur av lårbenshals) ?**

   - ☐ 1. Ja. (Hon var ungefär 4............år då.)
   - ☐ 2. Nej.
   - ☐ 3. Vet inte.

4. **Hur mycket mjölk, fil eller youghort får Du i Dig om dan ?**

   Jag dricker .... stycken glas mjölk eller tallrikar fil om dan.

5. **Hur många skivor ost brukar Du äta varje dag ?**

   Jag äter ungefär .... stycken skivor ost om dan.

6. **Hur många koppar kaffe brukar Du dricka varje dag ?**

   Jag dricker ungefär .... koppar kaffe om dan.

---

[1] meof8028-4-6-2Xenk-K-00-3-Emma-3.doc/010923

7. Röker Du numera?

- ❑ 1. Nej, jag har aldrig rökt.
- ❑ 2. Nej, men jag har rökt förut.
- ❑ 3. Ja

8. Vad är Din nuvarande vikt?

Jag väger .... kg.

9. Hur lång är Du nu ?

Jag är .... cm lång.

10. Kan Du resa Dig upp från en vanlig stol 5 gånger i följd - utan att hjälpa till med armarna? Pröva gärna.

- ❑ 1. Ja, jag kan resa mig upp 5 gånger i följd utan att hjälpa till med armarna.
- ❑ 2. Nej.

11. Har Du ramlat omkull de sista 12 månaderna ?

- ❑ 1. Nej.
- ❑ 2. Ja, en gång.
- ❑ 3. Ja, två gånger.
- ❑ 4. Ja, tre gånger.
- ❑ 5. Ja, fyra gånger.
- ❑ 6. Ja, mer än fyra gånger.

12. Har Du brutit något ben i kroppen efter att Du fyllt 50 år ?

- ❑ 1. Ja.
- ❑ 2. Nej → gå i så fall vidare till fråga 14.

13. Var har Du då brutit Dig ? (Här kan Du kryssa i flera rutor.)
Vid **vilken ålder** bröt Du dig (ungefär) ?

❑	1. Lårbenshals vänster sida vid ålder ....
❑	2. Lårbenshals höger sida vid ålder ....
❑	3. Handled / underarm vä. vid ålder ....
❑	4. Handled / underarm hö vid ålder ....
❑	5. Överarm vä vid ålder ....
❑	6. Överarm hö vid ålder ....
❑	7. Fotled vä vid ålder ....
❑	8. Fotled hö vid ålder ....
❑	9. Ryggkota ihoptryckt vid ålder ....
❑	10. Annat benbrott ....
❑	11. Vet ej.

14. Har du tagit kortisontabletter (Prednisolon eller Betapred) under mer än tre månader sammanlagt?

☐ 1. Ja.
☐ 2. Nej.

15. När upphörde Dina menstruationer ?

Jag var .... år när menstruationerna upphörde.

16. Har Du fött barn? I så fall – hur många barn?

☐ 1. Nej.
☐ 2. Ja, jag har fått 3 .... barn.

17. Ser Du bra ?

☐ 1. Ja. jag ser bra (med eller utan glasögon.)
☐ 2. Nej, jag ser inte bra (även om jag har glasögon.)

18. Är Du opererad för <u>utslitna</u> höftleder (artros)?
(Här kan Du fylla i två rutor)

☐ 1. Ja, på vänster höft.
☐ 2. Ja, på höger höft.
☐ 3. Nej.

19. Kommer någon av Dina föräldrar från ett annat land än de nordiska länderna ?

☐ 1. Ja.
☐ 2. Nej.

20. Boende (Här kan du kryssa flera rutor)

☐ 1. Ensamboende.
☐ 2. Sammanboende / bor med make.
☐ 3. Bor med annan släkting eller vän.
☐ 4. Bor på servicehus eller sjukhem.
☐ 5. Annat, nämligen ....

21. Får Du regelbunden hjälp i hemmet ?

☐ 1. Nej.
☐ 2. Ja, av anhörig eller bekant minst 1 gång per vecka.
☐ 3. Ja, av hemtjänst var dag.
☐ 4. Ja, av hemtjänst 2 - 5 gånger per vecka.
☐ 5. Ja, av hemtjänst 1 gång per vecka.
☐ 6. Bor på servicehus eller sjukhem och får hjälp vid behov.

22. Tar Du någon medicin dagligen ? (Även vitamintabletter, receptfri eller hälsokostmedicin räknas som medicin).

- ❑ 1. Nej.
- ❑ 2. Ja.

Om Du har några frågor eller funderingar så skriv gärna några rader här :

____

____

____

____

Skicka formuläret i bifogat kuvert (porto är betalt) till Distriktsläkare Daniel Albertsson, Vårdcentralen Vislanda, Gröna gatan 8-10, 340 30 Vislanda.

TACK FÖR DIN MEDVERKAN !
