# Supplementary material for: Reduced Risk of Recurrent Fragility Fractures After a Primary Care–Based Fracture Prevention Intervention: A 20-Year Non-Randomized Controlled Follow-Up Study in Women Aged 70–100
Source: Scand J Prim Health Care. 2025 Nov 6;44(1):1–16. doi: 10.1080/02813432.2025.2571929 (PMC12918357; doi:10.1080/02813432.2025.2571929)
Supplement: Walk Well BROCHURE.docx [file IPRI_A_2571929_SM4447.docx]

**Appendix 1. Fall Prevention Brochure: "Walk Well" (English translation)**

**Originally produced in Swedish as "Gå bra" (1998, revised 2015). Distributed in 2002 to all intervention participants.**

**WALK WELL**

**If you want to know more, contact your health center, home healthcare services, or call 1177 for information about:**

- Local walking or exercise groups
- Balance and strength training with a physiotherapist
- Anti-slip shoe grips or hip protectors
- How to further prevent fractures and falls

**Good Advice**

- A daily walk is beneficial. It strengthens your muscles and bones, reduces dizziness, and improves general fitness. During the summer months, sunlight exposure helps the skin produce vitamin D, which maintains bone strength.
- Simple exercises at home or in a group improve mobility and reduce the risk of falling. If you tend to fall, start training and make your home safer.
- Eat a balanced diet and at least one cooked meal a day, with plenty of fiber, vegetables, and fruit. Cheese and milk are rich in calcium. A daily need is covered by one glass of milk, a bowl of yogurt, and four slices of cheese. Low-fat milk (1.5% or less) is enriched with vitamin D and has just as much calcium as whole milk.
- Good spirits, body and mind go hand in hand. Pain or worry can affect your wellbeing. Don’t isolate yourself—seek pleasant company and activities to maintain your health.

**Self-Checklist**

Tick "YES" or "NO":

- I take a daily walk
- I train balance and strength at home every week
- I train balance and strength in a group every week
- I use reflectors when walking in low light or darkness
- I wear anti-slip shoes or grips during winter
- I walk better with aids (e.g., walker, poles, or cane with spike tip)
- Stairways in my home have good lighting, non-slip surfaces, and sturdy handrails

**Did You Know?**

- Falls are the most common cause of serious bodily injury.
- Hip fractures are common among older people, especially women.
- Fall-related injuries have increased due to aging and reduced physical activity.
- Most fall injuries happen at home due to tripping or slipping.
- Falls can be prevented by regular movement and reducing home hazards.
- If you fall often, you may need a health check. Fatigue, dizziness, or poor vision increase risk.
- If you have fallen before and broken a bone, using hip protectors can prevent hip fractures.
- Good physical condition helps faster recovery, even after serious injuries.

**More Self-Checks**

- I have non-slip mats in the tub, shower, and bathroom floor
- I have wall-mounted grab bars near the bath and toilet
- I sit down when showering
- I keep frequently used items within easy reach
- I avoid climbing on chairs or stools
- I have good lighting and a nightlight if I get up at night
- I have a phone near my bed or carry a mobile phone
- I use hip protectors because I fall often and have broken bones
- I eat a balanced, nutritious diet including cheese and milk

**Preventing Injuries at Home**

- Remove loose rugs from frequently used areas
- Use anti-slip underlays for rugs; replace them after a few years
- Wall-to-wall carpet with rubber backing is even safer
- Secure loose cords and cables
- Ensure sufficient lighting in all rooms

**More Safety Checks**

- I have removed unnecessary rugs
- I use anti-slip mats under rugs
- I wear sturdy indoor shoes with good soles or non-slip socks
- I have secured all loose cords
- I avoid floor polish to reduce slipperiness
- I have removed unnecessary thresholds in my home
- I have good lighting in all rooms
- I have had my vision checked and wear proper glasses
- I sit when dressing or undressing

**Daily Living Tips**

- Have your vision checked regularly (ideally once a year). Wear the right glasses!
- Always get up slowly from bed. If you get dizzy, sit a moment and move your feet on the floor first. Morning stretches in bed can help.
- Keep a phone within easy reach of your bed, or use a mobile phone.
- Use nightlights if you need to visit the bathroom at night.
- Sit down while dressing if you feel unsteady.

**If You Fall**

It’s good to know how to get up by yourself. Practice this with someone nearby who can help. If you fall:

1. Roll onto your side and get into a seated position.
2. Move to a sturdy piece of furniture (e.g., a chair or sofa) by crawling or sliding.
3. Use your strongest leg and arm to push yourself up while holding the furniture.
4. Sit down and rest.

If you can’t get up:

- Reach for a blanket to stay warm
- Make noise by knocking on the floor
- Always carry a mobile phone when possible

**Home Exercise Tips for Balance and Strength**

- A 30-minute walk daily is good exercise. Walk briskly and on uneven surfaces if possible. Walking with poles strengthens arms and straightens posture. Gymnastics, dancing, and gardening are also good.
- If you're unaccustomed to walking, start with short sessions. Even simple movements strengthen untrained muscles. Stamina often improves after 1–2 months.
- If outdoor walking is difficult, consider using an indoor exercise bike. Increase resistance slowly and train for 15–20 minutes daily.

**Balance Exercises (use a chair for support)**

- Stand with feet together for one minute, then try with eyes closed
- Repeat while standing on a foam pad
- Stand on one leg
- Rise up on toes 5–20 times
- Do deep knee bends
- Try the above without holding the chair when confident

Move slowly and mindfully. Focus on how your body feels during the exercises. Start daily for 1–2 months, then continue at least three times per week.

**Footwear and Aids**

- Wear well-fitting indoor shoes with firm heel support and good soles. Alternatives include non-slip socks.
- Never walk in plain socks.
- Remove unnecessary thresholds—check with your landlord or home care services first.
- Hip protectors can prevent fractures. If you fall often, talk to your healthcare provider.
- Use sturdy outdoor shoes. Anti-slip grips are essential in icy conditions.
- Use reflectors and walking aids such as rollators, poles, or canes with ice tips.

Anti-slip grips should fit securely, be easy to put on and remove, and cover the full sole. Remove them indoors on hard floors to avoid slipping.
